# The impact of cryosolution thermal contraction on proteins and protein crystals: volumes, conformation and order

**DOI:** 10.1107/S2059798318008793

**Published:** 2018-09-05

**Authors:** Douglas H. Juers, Christopher A. Farley, Christopher P. Saxby, Rosemary A. Cotter, Jackson K. B. Cahn, R. Conor Holton-Burke, Kaitlin Harrison, Zhenguo Wu

**Affiliations:** a Department of Physics, Whitman College, 345 Boyer Avenue, Walla Walla, WA 99362, USA; b Program in BBMB, Whitman College, 345 Boyer Avenue, Walla Walla, WA 99362, USA

**Keywords:** cryoprotection, thermal contraction, mosaicity, ice formation, cryocooling, crystal damage, optimization

## Abstract

Cryocooling of macromolecular crystals is commonly employed to limit radiation damage during X-ray diffraction data collection. However, cooling itself affects macromolecular conformation and often damages crystals *via* poorly understood processes. Here, the effects of cryosolution thermal contraction on macromolecular conformation and crystal order in crystals ranging from 32 to 67% solvent content are systematically investigated. It is found that the solution thermal contraction affects macromolecule configurations and volumes, unit-cell volumes, crystal packing and crystal order. The effects occur through not only thermal contraction, but also pressure caused by the mismatched contraction of cryosolvent and pores. Higher solvent-content crystals are more affected. In some cases the solvent contraction can be adjusted to reduce mosaicity and increase the strength of diffraction. Ice formation in some crystals is found to cause damage *via* a reduction in unit-cell volume, which is interpreted through solvent transport out of unit cells during cooling. The results point to more deductive approaches to cryoprotection optimization by adjusting the cryosolution composition to reduce thermal contraction-induced stresses in the crystal with cooling.

## Abstract 2

Cryosolution thermal contraction is found to have an impact on cryocooled protein and unit-cell volumes and conformations. In some cases, its adjustment can produce higher quality diffraction data.

## 1. Abbreviations and symbols   

DMSO: dimethyl sulfoxide.

MPD: 2-methyl-2,4-pentanediol.

EDTA: ethylenediaminetetraacetic acid.

R.m.s.d.: root-mean-square deviation.

SAD: single-wavelength anomalous diffraction.

LT: low temperature (100 or 77 K, depending on context).

RT: room temperature (294 K unless otherwise noted).

*V_x_* ^LT^, *V_x_* ^RT^: volume of *x* at LT or RT, where *x* is (unit) cell, chan(nel) or protein.

Δ_*x*_: the fractional volume change of *x* with cooling, (*V_x_* ^LT^ − *V_x_* ^RT^)/*V_x_* ^RT^.

ν_sol_: solvent content of the crystal at RT, *V* ^RT^_chan_/*V* ^RT^_cell_; often called the porosity.

ν_prot_: protein content of the crystal at RT, 1 − ν_sol_.

Δ^*T*^_sol_: intrinsic fractional change of the solvent specific volume with cooling from bulk measurements.

Δ^*T*^_sol,match_: value of Δ^*T*^_sol_ that matches Δ_chan_ for a given protein crystal.

*f*_bdy_, *f*_bulk_: volume fraction of the solvent in the crystal that is boundary or bulk solvent.

Δ^*T*^_sol,bdy_: fractional change of the boundary solvent volume with cooling.

β_sol_: thermal expansion coefficient of the solvent, Δ^*T*^_sol_ = 〈β_sol_〉Δ*T*.

β_chan_: thermal expansion coefficient of the channel or pore.

κ_sol_: isothermal compressibility of the solvent.

κ_chan_: isothermal compressibility of the channel or pore.

η: viscosity of the solvent.

ν_exit_: volume fraction of the solvent that exits the unit cell with cooling.

## 2. Introduction   

Diffraction data collection for macromolecular structure determination is commonly carried out at cryogenic temperature, which not only reduces the rate of radiation damage but also allows the kinetic trapping of intermediates for functional studies (Garman & Schneider, 1997 ▸; Weik & Colletier, 2010 ▸). The most common approach for cryo-mounting is to fish the crystal into a small nylon loop or micromount using surface tension and then to rapidly cool the crystal by plunging it into liquid nitrogen or directly transferring it to a nitrogen-vapour stream at ∼100 K (Teng, 1990 ▸; Thorne *et al.*, 2003 ▸).

However, the cryo-mounting process itself can damage crystals, increasing the mosaicity (crystal disorder) and reducing the diffraction power. Therefore, many crystals require some kind of treatment before or during cooling to limit cooling-induced damage. Most schemes are directed towards limiting ice formation in the sample by increasing the cooling rate, reducing the ice crystallization rate or cooling under high pressure (Thomanek *et al.*, 1973 ▸; Walker *et al.*, 1998 ▸; Thorne *et al.*, 2003 ▸; Kim *et al.*, 2005 ▸; Kitago *et al.*, 2005 ▸, 2010 ▸; Warkentin *et al.*, 2006 ▸; Warkentin & Thorne, 2009 ▸; Pellegrini *et al.*, 2011 ▸; Burkhardt *et al.*, 2012 ▸).

Although this approach is effective for many crystals, simply preventing ice formation is neither necessary nor sufficient for high-quality diffraction in all cases. Even when cooling conditions are found to be adequate for a particular purpose, they can often be further optimized by adjusting the concentration or the identity of the cryoprotective agent (Mitchell & Garman, 1994 ▸). Such optimization may be understood by considering the crystal as a composite material with a macromolecular component and a bulk-solution component contained within nanometre-sized pores that run throughout the crystal as well as coating the outer surface of the crystal (Kriminski *et al.*, 2002 ▸; Juers & Matthews, 2001 ▸, 2004*a* ▸,*b* ▸; Alcorn & Juers, 2010 ▸). The thermal behavior of such a system is complex, with potentially different thermal responses of the protein, internal solvent and external solvent. Cryosolution optimization then involves adjusting the solution contraction to limit the build-up of stresses during cooling. The average contractions of many different cryosolutions between RT and 77 K have been measured both in macroscopic samples (∼1 ml; Marshall *et al.*, 2012 ▸; Juers & Matthews, 2001 ▸, 2004*a* ▸; Alcorn & Juers, 2010 ▸) and in microlitre-sized drops (Shen *et al.*, 2016 ▸, 2017 ▸), but systematic testing of contraction-based cryo-optimization has not yet been reported.

There are several related questions about cryocooling that we aim to address here. How does the internal and/or external cryosolvent thermal contraction affect the thermal response of the crystal and the protein? Can the cryosolvent thermal contraction be adjusted to limit cooling-induced damage? What role does the solvent content of crystals play? Exactly how does ice formation damage crystals?

To address these questions, here we measure X-ray diffraction from nine different protein crystals with a range of pore sizes equilibrated with cryosolvents with a range of contractions. We find that the crystal thermal response depends strongly on the crystal pore size. Solvent contraction can impact the contraction of the crystal, the conformation of the protein and the interactions between proteins in the crystal. In some cases the internal cryosolvent contraction can be optimized to reduce the mosaicity, but the external cryosolvent contraction seems to have modest effects at most. Exposing crystals to a range of solution contractions can be employed to explore protein conformational variability. The results support rational approaches for cryocooling optimization based on knowledge of the crystal solvent content and intrinsic thermal contractions of crystal solutions.

## 3. Materials and methods   

### 3.1. Chemicals   

All proteins and crystallization reagents and most of the cryoprotective agents were purchased from Sigma–Aldrich. Protein catalog numbers are as described previously (Farley & Juers, 2014 ▸). Immersion Oils A, B and NVH were from Cargille Laboratories (Cedar Grove, New Jersey, USA), Paratac and Infineum V8512 were from Sea–Land Chemical Company (Westlake, Ohio, USA), Fomblin YR1800 from Alfa–Aesar (Haverhill, Massachusetts, USA) and paraffin oil from Mallinckrodt (VWR Scientific Products). All cryoprotective agent concentrations are reported as percentage (*w*/*w*).

### 3.2. Crystals   

Crystals were grown using hanging-drop vapor diffusion in 24-well plates at 294 K (RT). Triclinic lysozyme crystals were grown using 10 mg ml^−1^ protein against 0.3 *M* NaNO_3_, with microseeding yielding the largest crystals. α-Lactalbumin crystals were grown using 30–50 mg ml^−1^ protein against 50 m*M* KH_2_PO_4_, 15–20% PEG 8000 (Mueller-Dieckmann *et al.*, 2007 ▸). The other crystals were grown as described previously (Farley & Juers, 2014 ▸). Crystal sizes were as follows: tetragonal thermolysin, 300–400 µm octohedra; cubic insulin, 200–600 µm cubes; tetragonal thaumatin, 200–400 µm octohedra; hexagonal thermolysin, 100–400 µm rods; ortho­rhombic trypsin, 200–500 µm parallelpipeds; tetragonal lysozyme, 200–400 µm parellelpipeds; trigonal trypsin, 150–200 µm chunks; triclinic lysozyme, 30–100 µm chunks; α-lactalbumin, 200–800 µm rectangular parallelpipeds.

### 3.3. Cryoprotection and crystal mounting   

All crystals were manipulated and soaked under humid flow at RT (294 K; Farley *et al.*, 2014 ▸). A relative humidity (RH) value of ∼90% was employed, which is between the minimum and maximum RH values of the solutions used. Manipulations and mounts were performed with cryoloops (Hampton Research, Aliso Viejo, California, USA) that were about the same size as the crystal, with some contact between the loop and the crystal in most cases. Crystals were transferred to 15–30 µl drops of cryosolution (RT) in one step if possible, or else were serially soaked to the target cryoprotectant concentration in 2–5 steps with increasing concentration as necessary to prevent cracking. Cryoprotective agents not only inhibit the formation of ice, but also modulate other solution properties, including the intrinsic solution contraction. At 50%(*w*/*w*), common cryosolutions show contractions that range from very small (for example ∼1%) to greater than 10%. Here, the goal was to investigate crystal properties as a function of cryo­solution contraction, which was accomplished by equilibrating the crystals with a range of cryosolutions. Two approaches were used to achieve a wide range of contractions. Firstly, binary mixtures of a high contractor (*i.e.* MPD, 8.5%) and a low contractor (*i.e.* xylose, 3.0%) were used. Secondly, a range of cryoprotective agents from Table 1 of Alcorn & Juers (2010 ▸) were used. For contractions smaller than 3.0%, lower concentrations of xylose were used. In most cases, crystals were equilibrated with pure cryosolution. For triclinic lysozyme, tetragonal lysozyme and some cubic insulin crystals, the cryosolution supplemented a stabilizing solution (0.3 *M* NaNO_3_, 5% NaCl and 20 m*M* sodium phosphate pH 9.2/0.2 m*M* EDTA, respectively; see Supplementary Table S1). In each case where 50% ethanol (EtOH) was used (ortho­rhombic trypsin, hexagonal thermolysin and tetragonal thermo­lysin), powder rings were observed consistent with neither ice I_h_ nor ice I_c_, but with a type I or modified type I clathrate [space group 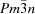, *a* = 11.97 (0.01) Å; Facq *et al.*, 2013 ▸]. Information about these 50% ethanol data sets is included in Supplementary Tables S1, S6 and S7, but is omitted from the plots. Similar rings were not observed when 50% methanol (MeOH) was used.

Crystals were cryocooled in a cryostream using the vial-mounting method as described previously (Farley *et al.*, 2014 ▸). Briefly, cryovials (Hampton Research) were prepared by plugging the liquid-nitrogen escape holes with clay and pipetting 500 µl of the cryosolution into the vial. Crystals were then mounted into loops and the crystal cap was placed in the cryovial (Hampton Research). The crystal was then allowed to vapor-equilibrate in the vial with the cryosolution in the bottom of the vial. After the in-vial equilibration, the crystals were directly mounted from the vial onto the diffractometer in a cryostream (Cryojet, Oxford Instruments, Oxford, England). This approach limits both the random and systematic errors associated with dehydration during transfer to the cooling medium. The cold-stream flow rates were set at 6 l min^−1^ (sample) and 4 l min^−1^ (shell) and the sample-flow temperature was set to 100 K (LT).

The soak times were 1–3 min and the vial times were 15 s to 3 min, both at RT. For hexagonal thermolysin crystals, the soak and/or vial times affected the mosaicities and the cell volumes for the highly contracting solvents [*i.e.* MeOH, dimethylformamide (DMF) and MPD]. A range of times was therefore explored for these solvents.

For room-temperature experiments, crystals were mounted in MicroRT tubes (MiTeGen, Ithaca, New York, USA) or in glass capillaries (Hampton Research). In some cases the crystals were not stable at RT in the cryosolution on the timescale of data collection (minutes to hours). In these cases cell volumes were extrapolated back to the beginning of the data set or the pre-experiment values were used (*i.e.* 50% MPD, 50% xylose for trypsin; see Supplementary Table S1).

For triclinic lysozyme, the variation in cell contraction was reduced by using the same crystal for RT and LT data collection. After RT data collection in the MicroRT tube, the crystal was unmounted into a vial while under humid flow and then remounted at LT as described above after a 3 min vial equilibration. In all other cases different crystals were used for RT and LT data collection.

### 3.4. Solution contractions   

Cryosolvent contractions with cooling between 295 and 77 K (Supplementary Table S2) were determined either from previously published data (Juers & Matthews, 2004*a* ▸; Alcorn & Juers, 2010 ▸) or by direct measurement using a buoyancy-based density measurement with liquid nitrogen as the displaced liquid (Supplementary Table S3; Juers & Matthews, 2004*a* ▸; Alcorn & Juers, 2010 ▸). Contractions for some solutions were interpolated or extrapolated as follows. For xylose/water and xylose/MPD solutions, linear interpolations were performed between 50% xylose and water or 50% xylose and 50% MPD. For glycerol/water, MPD/water and DMSO/water solutions, Fig. 4 of Juers & Matthews (2004*a* ▸) was used. In cases where ice formed, interpolation (or extrapolation) was performed from the left (following the linear segment from 0% cryoprotective agent). In cases of vitrification, the interpolation (or extrapolation) was performed from the right, following the curved segment from 100% cryoprotective agent. For the glycerol solutions, the vitrified contraction values were cross-checked with those of Shen *et al.* (2016 ▸)

For four crystals, measurements were made to test the effects of the external cryoprotectant only by keeping a constant aqueous internal cryoprotectant and using different external oils with a range of contractions (11.3–5.0% contraction). The crystals and their internal cryoprotectants were triclinic lysozyme [23.5%(*w*/*w*) xylose], trigonal trypsin (well solution), hexagonal thermolysin [50%(*w*/*w*) xylose] and tetragonal thermolysin [50%(*w*/*w*) glucose].

### 3.5. X-ray data collection and processing   

X-ray data were collected using an Oxford Diffraction Xcalibur X-ray diffractometer with a Nova X-ray source and an Onyx detector (Rigaku Americas, The Woodlands, Texas, USA) at 50 kV and 0.8 mA. The beam divergence was 5.2 mrad (0.30°). Crystal-to-detector distances were in most cases 60–70 mm. In each case a pre-experiment was conducted with 2 × 6 (0.25 or 0.5°; 20–40 s) frames separated by 90°. The pre-experiment outputs estimates of cell parameters and mosaicities. In most cases, a full data set was then collected and integrated in *CrysAlis^Pro^* (Agilent, Yarnton, England), yielding post-refined crystal parameters. For the α-lactalbumin data collection the detector was set at 75 mm and 0.5° 60–90 s exposures were used, and in each case a complete data set was collected. In *CrysAlis^Pro^* the ‘mosaicity’ is given as three components, *e*_1_, *e*_2_ and *e*_3_, which are the mosaicities in three directions defined in a coordinate system local to each reflection. *e*_1_ and *e*_2_ are the mosaicities (*i.e.* the angle subtended by the diffraction spots) in two orthogonal directions tangential to the Ewald sphere (on the image, *e*_2_ is the mosaicity along the direction radial from the beam centre), while *e*_3_ is the mosaicity in a direction perpendicular to *e*_1_ and *s* − *s*_0_, which is roughly the mosaicity in the scanning direction, where *s* and *s*_0_ are the scattered and incident X-ray vectors, respectively (Kabsch, 2001 ▸). The *e*_3_ mosaicity parameter is similar to the REFLECTING_RANGE parameter in *XDS*. For the crystals tested here, the *e*_3_ values are about six times greater than the REFLECTING_RANGE_E.S.D., which is the value reported by *XDS* as the mosaicity (Kabsch, 2010 ▸). Data were integrated and scaled in *CrysAlis^Pro^* and merged using *SCALA* (Evans, 2006 ▸) and *CTRUNCATE* (French & Wilson, 1978 ▸; Winn *et al.*, 2011 ▸). The crystal order metrics used were the *e*_3_ mosaicity (*CrysAlis^Pro^*) and the Wilson *B* factor (*CTRUNCATE*). Powder diffraction patterns were analysed with *JADE* (MDI, Livermore, California, USA).

### 3.6. Structure determination and analysis   

Structures were determined in *PHENIX* (Adams *et al.*, 2010 ▸) with model building in *Coot* (Emsley *et al.*, 2010 ▸). For tetragonal thermolysin, SAD was used to solve the structure of the 40% xylose soak at LT (PDB entry 5uu9). The anomalous scatterers included four Ca^2+^ ions, two Zn^2+^ ions and two methionine S atoms. This structure was then used as a starting model for molecular-replacement solutions of the other tetragonal thermolysin structures. For the orthorhombic trypsin structures, the 50% xylose soak at LT was determined using molecular replacement starting with PDB entry 4i8h (an atomic resolution structure; Liebschner *et al.*, 2013 ▸). The resulting structure (PDB entry 6b6q) was then used as the starting point for molecular replacement of the other ortho­rhombic trypsin structures. For the hexagonal thermolysin structures, the starting model for the 50% MPD soak was a 1.25 Å resolution tetragonal thermolysin structure (unpublished work), which was then used as the starting model for the other structures. For *P*1 lysozyme, the starting model for one RT structure was PDB entry 4lzt (Walsh *et al.*, 1998 ▸), which was then used as the starting model for the other structures. In all cases, H atoms were included in the model in order to achieve more precise volume calculations. Protein volumes were calculated with *MSROLL* using a probe radius of 1.5 Å (Connolly, 1983 ▸). Pore diameters were calculated with *MAP_CHANNELS* (Juers & Ruffin, 2014 ▸). General manipulations, overlays and calculation of crystal contacts were performed with *EdPDB* (Zhang & Matthews, 1995 ▸). See Supporting Information for data collection and refinement statistics.

## 4. Results   

### 4.1. Cryosolvent contraction impacts unit-cell and protein volumes   

To investigate the effects of cryosolvent thermal contraction, tests were conducted with cryosolvents ranging from 13% contraction to 7% expansion. Throughout, the intrinsic bulk-solvent contraction is characterized by the fractional change in solvent specific volume with cooling, Δ^*T*^_sol_. To ensure that the observed effects were owing to the temperature change, care was taken to limit dehydration during crystal manipulation and transfer to the cryostream (see §3). LT cell parameters were determined from full X-ray data sets for each condition. RT cell parameters were based on averages of full data sets collected with a subset of the cryosolvents.

**Figure 1:**
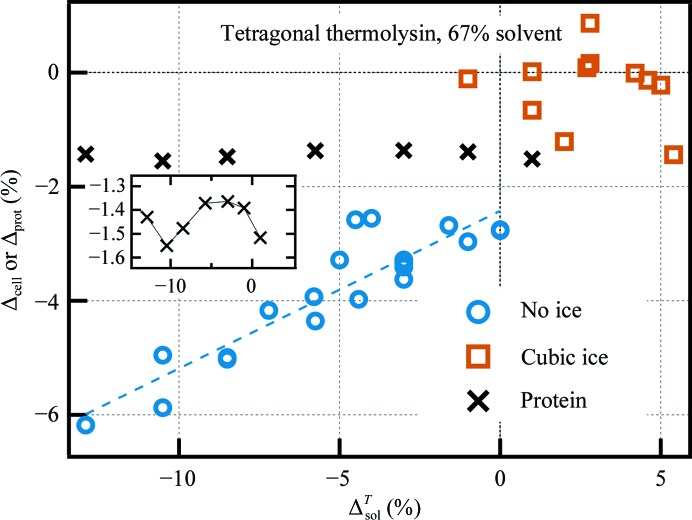
Cell and protein contractions with cooling for tetragonal thermolysin. Crystal parameters were measured for four RT conditions and ∼20 LT conditions. Cell-volume contractions were then calculated according to Δ_cell_ = (*V* ^LT^_cell_ − 〈*V* ^RT^_cell_〉)/〈*V* ^RT^_cell_〉, with the average taken over the four RT samples measured. An analogous calculation was performed for protein volume contractions, with protein volumes determined for two RT conditions and seven LT conditions. Dashed lines are fits of Δ_cell_ = ν_prot_Δ_prot_ + (1 − ν_prot_)Δ_chan_, with Δ_chan_ given by (2). The horizontal axis plots the fractional change in specific volume of solvent with cooling: Δ^*T*^_sol_ ≡ (ν_77 K_ − ν_294 K_)/ν_294 K_ = 〈β〉Δ*T*, where ν_77 K_ and ν_294 K_ are specific volumes based on density measurements in bulk (Alcorn & Juers, 2010 ▸), 〈β〉 represents the average thermal expansivity of the solution and Δ*T* = −217 K.

Fig. 1 ▸ shows the calculated volume contractions for tetragonal thermolysin (Supplementary Table S1). Greater solvent contractions yield greater cell contractions. The range of cell contractions (0–6%) roughly brackets the range of cell contractions in previously reported surveys (Juers & Matthews, 2001 ▸; Fraser *et al.*, 2011 ▸) and is linear with Δ^*T*^_sol_, especially for the cases in which the solvent vitrified.

Protein volumes were calculated with *MSROLL* using the refined structures (Connolly, 1983 ▸). As shown in Fig. 1 ▸, thermo­lysin contracts by 1.4–1.6%, depending on the solvent. The protein contracts the least for intermediate-contracting cryosolvents. As with the cell contraction, these protein contractions are similar to previously reported values.

**Figure 2:**
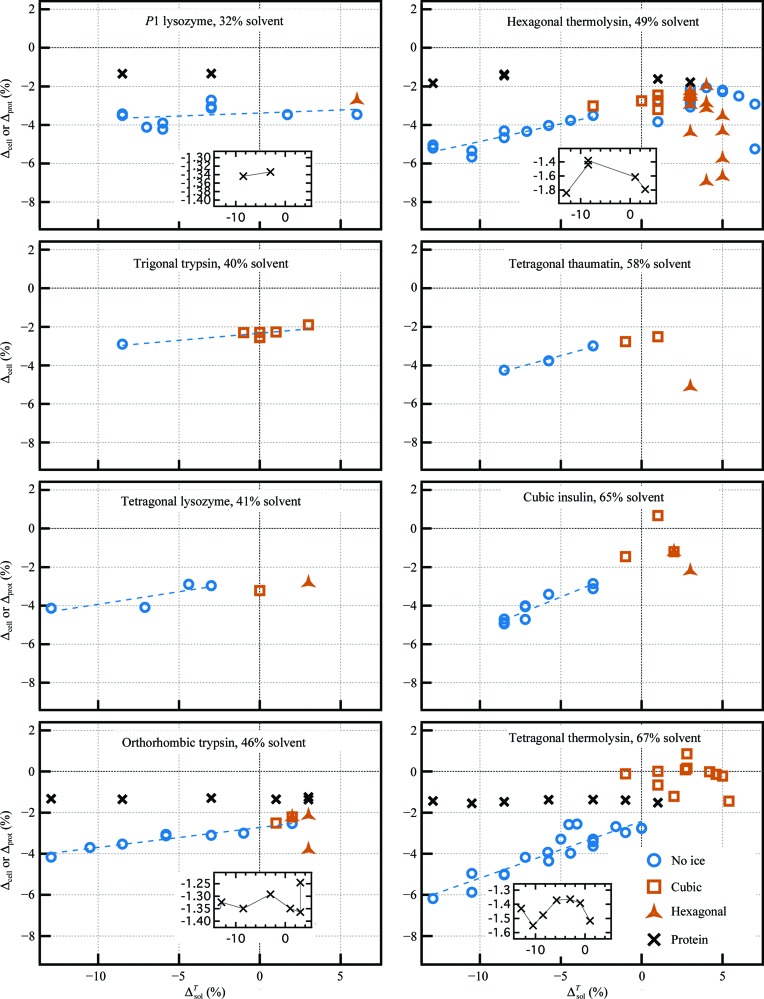
Crystal and protein contractions for all crystals tested. In five cases, RT cell volumes were determined by averaging over 1–4 RT conditions (Supplementary Table S1). For insulin and hexagonal thermolysin, linear and quadratic fits of *V*_cell_ *versus* Δ^*T*^_sol_ were used, respectively. For triclinic lysozyme, RT cell volumes were determined for each condition. In three cases, RT protein volumes were determined by averaging over 1–4 conditions. For triclinic lysozyme, RT protein volumes were determined for each of the two conditions. See the caption to Fig. 1 for an explanation of the dashed lines.

The behavior of the cryosolvent within the pores may depend on the size of the solvent channels, since the confinement of liquids to nanometre-sized pores strongly affects their properties (Teixeira *et al.*, 1997 ▸; Rasaiah *et al.*, 2008 ▸). Other protein crystals with a range of pore radii (5.5–20 Å; 32–67% solvent) were therefore investigated (Fig. 2 ▸). In general, the unit-cell contraction effects noted for tetragonal thermolysin become less pronounced for crystals with smaller pore sizes. Structures were also determined for a subset of the other proteins (triclinic lysozyme, orthorhombic trypsin and hexagonal thermolysin). Protein contraction values were 1.2–1.8% over the whole range of cryosolutions, with intermediate solution contractions yielding smaller protein contractions.

The effects on cell volume illustrated in Figs. 1 ▸ and 2 ▸ could be owing to contraction of the cryosolvent within the solvent channels (internal) and/or the solution coating the outside of the crystal (external). To separate these possibilities, we kept the internal cryosolvent constant while using a series of external oils with contractions ranging from 5.0 to 11.3%. There was no measureable dependence of the cell-volume contraction on the oil contraction (Δ_cell_ *versus* Δ^*T*^_sol_ slope = 0.002 ± 0.01 for tetragonal thermolysin; Supplementary Fig. S1*a*). Additionally, other tests were performed with tetragonal thermolysin. (i) Using just NVH oil, we used a range of loop sizes to vary the thickness of the external solution. This also had little impact on the cell volume or mosaicity, but crystals with thicker layers of oil showed slightly lower *I*/σ(*I*) values (Supplementary Table S1). (ii) The properties of crystals coated with NVH oil were still sensitive to the internal cryoprotectant. DMF-soaked crystals coated in NVH oil still showed greater cell contraction and higher mosaicity than xylose-soaked crystals coated in NVH oil (Supplementary Table S1). Together, these observations suggest a dependence of Δ_cell_ on bulk properties of the internal and not the external cryosolvent.

### 4.2. Cryosolvent contraction has an impact on protein conformation and crystal packing   

**Figure 3:**
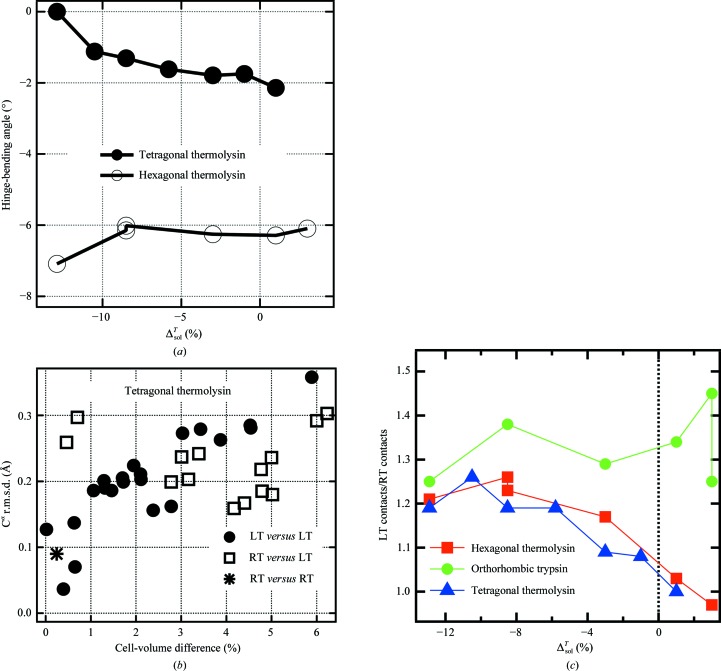
Protein conformation and crystal packing. (*a*) Dependence of the LT hinge-bending angle between the N-terminal and C-terminal domains of thermolysin on cryosolution contraction. The reference state is tetragonal thermolysin with methanol as a cryoprotectant. More negative values correspond to more ‘closed’ conformations. Domain definitions from Holland *et al.* (1992 ▸) were used. The RT values for tetragonal thermolysin are −1.9 and −1.8° (for MPD and xylose; Δ^*T*^_sol_ = −0.085 and −0.030, respectively). This RT effect accounts for ∼30% of the dependence of the tetragonal thermolysin hinge-bending angle on cryosolvent contraction, suggesting that the remainder is owing to differential contraction. RT values for hexagonal thermolysin are −6.0 and −5.9° (for 50% DMF and 50% xylose; Δ^*T*^_sol_ = −0.105 and −0.030, respectively), over the region of little change in the hexagonal thermolysin hinge-bending angle. (*b*) Dependence of structural difference (C^α^ r.m.s.d.) on unit-cell difference for tetragonal thermolysin. If the cell volumes are similar, two LT structures can be as similar as two RT structures. However, increasing cell-volume difference correlates with larger LT structural difference, so that two LT structures can be as different as an LT and an RT structure. (*c*) The relationship between crystal contacts and solvent contraction. Crystal contacts were calculated using a uniform 4.0 Å centre-to-centre distance cutoff for all atoms using *EdPDB* (Zhang & Matthews, 1995 ▸). All LT structures were compared with the 50%(*w*/*w*) RT xylose soaks. In most cases, cooling increases the number of crystal contacts. For the thermolysins, greater solvent contraction increases the number of crystal contacts relative to room temperature, while this trend is not obvious for orthorhombic trypsin. All LT structures with positive values of Δ^*T*^_sol_ showed some ice formation. Note that the highest ratio for trypsin occurred with the greatest cell reduction at 20% xylose with ice formation. Other levels of stringency for calculating contacts showed qualitatively similar results (Supplementary Fig. S3).

Protein conformation was investigated in two ways. Firstly, thermolysin is known to show a hinge-bending variation, which is thought to be related to substrate binding (Holland *et al.*, 1992 ▸). A change in the hinge-bending angle of 5° was observed on comparing the structures from two different crystal forms of thermolysin (Hausrath & Matthews, 2002 ▸). Here, we observed a 2° range in the hinge-bending angle for tetragonal thermolysin, which was highly correlated with the cryosolvent contraction, as shown in Fig. 3 ▸(*a*). Secondly, all pairs of tetragonal thermolysin structures from different cryosolvents were superimposed and the r.m.s.d.s of C^α^ positions were calculated. Fig. 3 ▸(*b*) shows that there is a correlation between the r.m.s.d. and the cell-volume difference for the LT structures (correlation coefficient of 0.87). Hexagonal thermolysin and orthorhombic trypsin showed similar behavior (Supplementary Fig. S2).

Cooling increases the number of atomic contacts between proteins within the crystal (Frauenfelder *et al.*, 1987 ▸; Juers & Matthews, 2001 ▸; Fraser *et al.*, 2011 ▸). We calculated the ratio of the number of contacts at LT compared with RT using three levels of stringency (Fig. 3 ▸ *c* and Supplementary Fig. S3). The plots suggest that for tetragonal and hexagonal thermolysin the increase in contacts is greater for higher contracting solvents, but this trend is not obvious for orthorhombic trypsin.

### 4.3. Crystal order, thermal contraction and ice formation   

**Figure 4:**
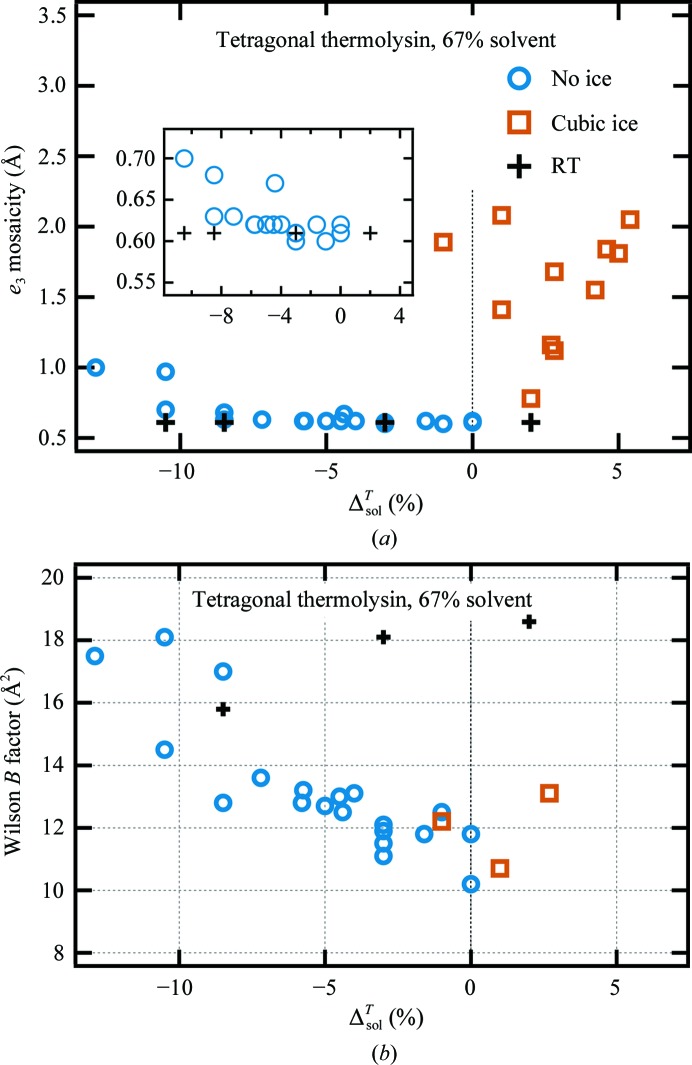
Crystal order and diffraction properties as a function of cryosolution contraction for tetragonal thermolysin. (*a*) *e*_3_ mosaicity. (*b*) Wilson *B* factor.

To investigate crystal order, two metrics were used: mosaicity and the overall *B* factor (Wilson *B* factor). For tetragonal thermolysin, cryosolvents with greater contractions correlate with higher mosaicities and Wilson *B* factors (Fig. 4 ▸). At RT, the overall *B* factor is 16–18 Å^2^. Cooling reduces this to as low as 10 Å^2^, but the highest contracting cryosolvents produce overall *B* factors similar to those at RT. Reducing the pore size reduces the correlation between cryosolvent contraction and crystal order (Supplementary Figs. S4 and S5).

As the cryosolvent was adjusted to small contractions and then small expansions (*i.e.* moving from left to right in Fig. 4 ▸ and Supplementary Fig. S4) three notable effects occurred at some point. Firstly, the mosaicity increased sharply and the diffraction limit plummeted. Secondly, ice rings were observed in the diffraction pattern: first cubic ice I_c_ and then hexagonal ice I_h_ at lower concentrations of cryoprotective agent. Thirdly, for intermediate pore sizes changing to a cryosolvent with less contraction (or greater expansion) actually caused the cell to contract more (Figs. 1 ▸ and 2 ▸). Attempts were made to separate out effects from ice formation *versus* effects from thermal contraction, with hexagonal thermolysin, which is remarkably permissive of low cryoprotectant concentrations, being stable in pure water for short times. Here, ice formation visible in the diffraction pattern was eliminated by removing the aqueous external cryosolution. This was achieved by using an external oil and removing the remaining external aqueous cryosolution away from the crystal surface with a fine micropipette, or by using long crystals that extended far from the mounting loop and blotting the crystal under humid flow, which left an essentially naked region that would usually cool without ice formation. With these steps, mosaicities were reduced from their values with ice, but not to the minimal levels observed at higher concentrations of cryoprotective agents (Supplementary Fig. S4). Diffraction was still relatively poor for the lowest cryosolvent concentrations (*i.e.* diffraction to ∼10, 3 and 2.5 Å for pure water, 10% xylose and 20% xylose, respectively). The unit-cell contractions were also less dramatic, but the cell still contracted more as the cryoprotective concentration was reduced (Fig. 2 ▸).

### 4.4. Case study: α-lactalbumin   

Most of the crystals described thus far are well characterized and well diffracting crystals. To test the cryo-optimization concepts presented here, we used a less well characterized crystal. There are four structures of uncomplexed *Bos taurus* α-lactalbumin (123 residues) in the Protein Data Bank, all based on X-ray data collected at synchrotrons to resolutions of 2.2–2.3 Å. Just one of these is at 100 K, with high mosaicity (1.0° in *DENZO*/*SCALEPACK*; Mueller-Dieckmann *et al.*, 2007 ▸). We therefore set out to optimize cooling of this crystal using thermal contraction as a guide. The crystal form used was an orthorhombic crystal form (*P*2_1_2_1_2) with RT unit-cell parameters *a* = 72.0, *b* = 104.7, *c* = 117.4 Å (PDB entry 1f6s; Chrysina *et al.*, 2000 ▸), six molecules per asymmetric unit, 53% solvent content and a maximum pore radius of 12.2 Å.

**Figure 5:**
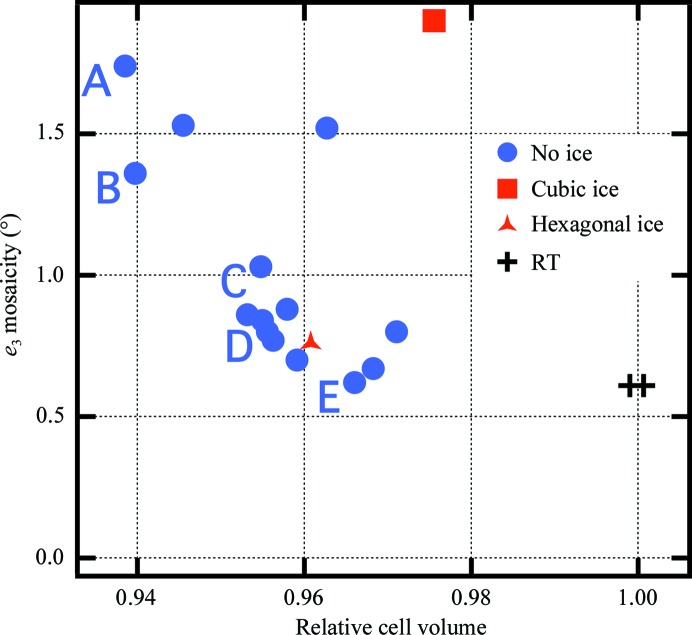
Relationship between cell volume and mosaicity for α-lactalbumin crystals. The cell volumes are normalized to the two room-temperature values on the right. All other points are from 100 K data. The letters (A–E) label crystals for which cryoconditions during cryo-optimization are described in the text. There appears to be an optimal cell volume yielding the lowest mosaicity at about Δ_cell_ ≅ −3.5%.

We first started with glycerol and, as shown in Fig. 5 ▸, found a very high mosaicity of 1.7° (A in Fig. 5 ▸) at 25% glycerol with diffraction to about 2.8 Å resolution [using 〈*I*/σ(*I*)〉 = 1 in the high-resolution bin to define the resolution limit]. Since this solution should already have a relatively low contraction, we tried increasing the contraction by using first MPD and then vapor diffusion of MeOH (Farley *et al.*, 2014 ▸), which improved the diffraction. At 35% MeOH the mosaicity was reduced to 1.4° (B in Fig. 5 ▸), with diffraction to about 2.2 Å resolution. To make sure that it was reasonable to expect further improvement, we then checked a room-temperature crystal, finding a mosaicity of 0.6° with diffraction to about 2.2 Å resolution. Higher concentrations of MeOH produced apparent precipitation in the crystal, so we soaked the crystals in low salt (10 m*M* KH_2_PO_4_). Under low-salt conditions and 40% MeOH, the mosaicity was reduced to 1.0° (C in Fig. 5 ▸). Higher concentrations of MeOH were not tolerated, so we then tried reducing the contraction. At 35% MeOH the mosaicity decreased to 0.8° (D in Fig. 5 ▸), but lower MeOH concentrations caused ice formation. The crystals were susceptible to cracking during soaks, so to achieve a lower contraction without too much soaking we soaked the crystal in 10% MPD (at low salt) and then vapor-equilibrated to 25% MeOH (E in Fig. 5 ▸). This prevented ice formation and yielded a mosaicity of 0.62° with diffraction to 1.7 Å resolution. Fig. 5 ▸ shows the relationship between cell volume and mosaicity for the crystals from which we collected full data sets. Later, we found serials soaks to 25% xylose also yielded low mosaicity (0.67°), but lower resolution data (about 2.0 Å) and a much higher Wilson *B* factor (36 Å^2^, compared with 18 Å^2^ for the MPD/MeOH-soaked crystal; the RT crystal Wilson *B* factors were ∼38 Å^2^). For further details, see Supplementary Table S12.

## 5. Discussion   

### 5.1. Volume changes of the unit-cell components with cooling and contraction of cryosolvent   

Cooling for X-ray data collection reduces the volume of the protein and, to a greater degree, the unit cell (Frauenfelder *et al.*, 1987 ▸; Juers & Matthews, 2001 ▸; Fraser *et al.*, 2011 ▸). It can be readily shown that the fractional changes in the volumes of the cell, protein and channels with cooling are related by Δ_cell_ = (1 − ν_sol_)Δ_prot_ + ν_sol_Δ_chan_, where ν_sol_ is the room-temperature solvent content. The channel contraction can therefore be expressed as
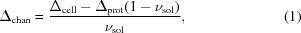
which gives values ∼2–11 times greater than the protein contractions for the crystals tested here (Supplementary Table S1). This is corroborated by direct calculation of channel diameters from coordinates using *MAP_CHANNELS*, which shows the largest channel diameter of tetragonal thermolysin to contract by up to 5%, while the protein radius of gyration contracts by less than 1% (Supplementary Fig. S6).

Greater contraction of the channels than of protein implies that the protein matrix is not completely rigid and that its thermal response is not a simple uniform size scaling, but may involve conformational changes within the protein and the remodeling of intermolecular contacts, as has been discussed by Juers & Matthews (2001 ▸). This results in a modulation of the shape of the protein matrix as well as its contraction to yield (in most cases) greater contraction of the channels than the protein matrix. Here, to gain further insight, we consider how the contractions of the crystal components depend on the intrinsic solvent contraction.

**Figure 6:**
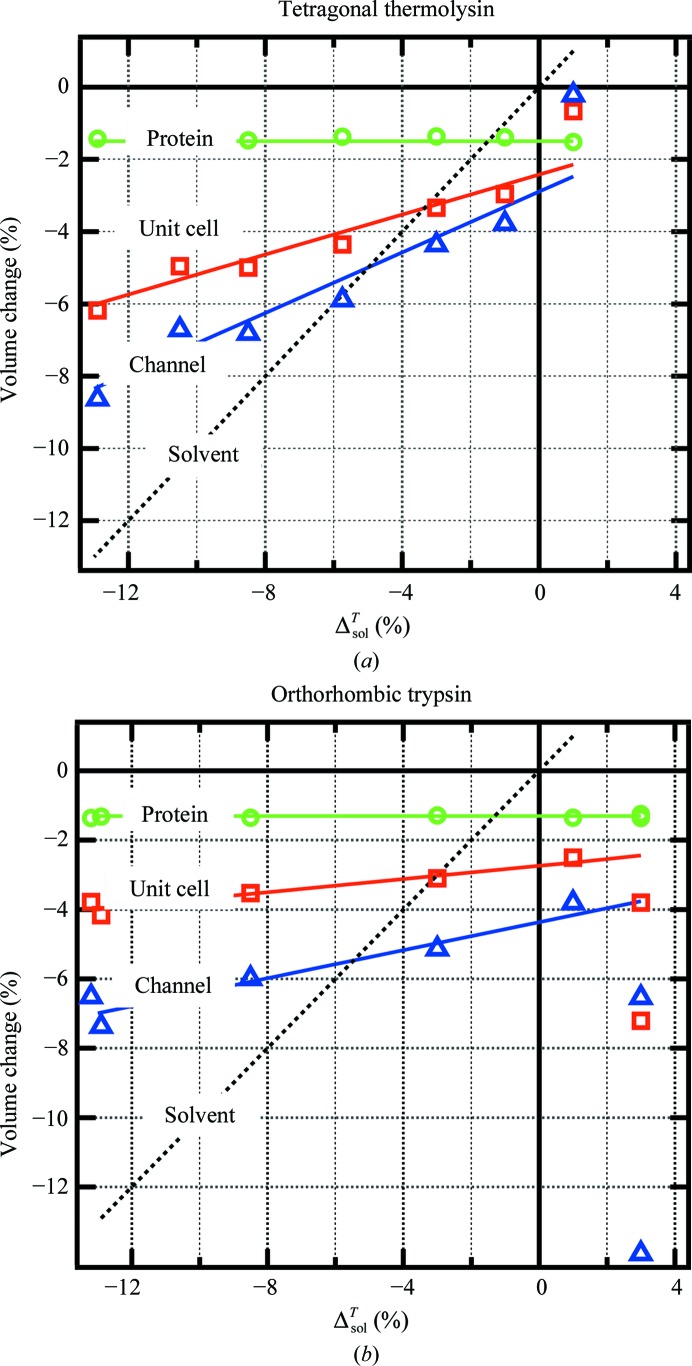
Schematics showing the dependence of the cooling response of the unit-cell components on cryosolvent contraction for (*a*) tetragonal thermolysin and (*b*) orthorhombic trypsin. Cell and protein contractions were calculated as described in Fig. 1 ▸. The channel contraction was calculated according to Δ_chan_ = (Δ_cell_ − ν_prot_Δ_prot_)/(1 − Δ_prot_). The lines shown are linear fits to the data over most of the range.

Fig. 6 ▸ shows schematics of the cooling-induced volume changes of the unit-cell components *versus* Δ^*T*^_sol_ for tetragonal thermolysin and orthorhombic trypsin. There is one value of Δ^*T*^_sol_ which matches Δ_chan_, which we call Δ^*T*^_sol,match_ (−0.050 and −0.055 for thermolysin and trypsin, respectively). The other crystals show similar schematics, with Δ^*T*^_sol,match_ values ranging from −0.085 to −0.045 (Table 1 ▸). There are several features of the plots in Fig. 6 ▸ that we seek to understand: the mismatch between Δ_chan_ and Δ^*T*^_sol_ over most of the range, the smaller dependence of Δ_chan_ on Δ^*T*^_sol_ for low solvent-content crystals, and the departure from linearity of Δ_chan_ and Δ_cell_ *versus* Δ^*T*^_sol_ for some expanding solvents.

We attribute the observed effects to the temperature change. However, water and other solution components can evaporate during crystal mounting, shrinking cell volumes (Farley *et al.*, 2014 ▸). Here, we employed techniques to limit evaporation, which reduced noise and the systematic underestimation of LT cell volumes and also allowed effective cooling with alcohols (Farley & Juers, 2014 ▸).

#### 5.1.1. Modulation of solvent thermal contraction by the channels   

We first consider the possibility that the intrinsic thermal contractions of the channels and solvent are actually matched over the whole range owing to solvent confinement. The thermal properties of solvent in the pores of protein crystals are most likely modulated by contact with the protein surface as well as being restricted to a small volume. For example, water confined in porous silica shows a reduced freezing point owing to the Gibbs–Thomson effect: to 235 K in 21 Å radius pores and to <130 K in 7.5 Å radius pores (Findenegg *et al.*, 2008 ▸; Liu *et al.*, 2013 ▸). It is also proposed to have an elevated density and thermal expansion in the 6 Å boundary layer adjacent to the pore wall (Xu *et al.*, 2009 ▸).

The preferential exclusion of cosolvents from the protein surface may also play a role (Timasheff, 2002*a* ▸,*b* ▸; Auton *et al.*, 2011 ▸; Shen *et al.*, 2016 ▸). In comparison to water molecules, we observe five to ten times fewer cryoprotective agents in our electron-density maps than would be expected for concentrations of 50%(*w*/*w*) (Supplementary Tables S5, S7, S9 and S11). Thus, the actual composition of the boundary solvent may not be very different for the various cryosolvents.

**Figure 7:**
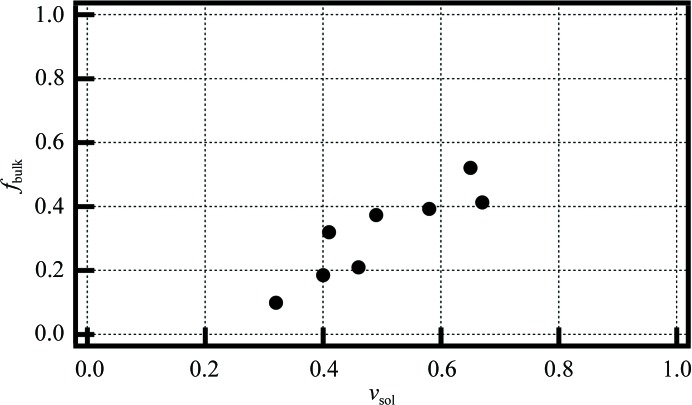
‘Bulk factor’, *f*_bulk_, *versus* solvent content. *f*_bulk_ is a fitting parameter (see §[Sec sec4.2]4.2) reflecting the fraction of solvent within the pores that shows bulk contraction values. Each plotted point represents one of the eight protein crystals tested. Greater solvent content is correlated with a greater fraction of solvent within the channels behaving as bulk.

A simple model to account for the confined behavior then divides the channel into a boundary component (fraction *f*_bdy_) with constant contraction Δ^*T*^_sol,bdy_ and a bulk component (fraction *f*_bulk_ = 1 − *f*_bdy_) with our measured contraction, Δ^*T*^_sol_, 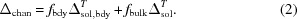



Fits of this model (dashed lines in Fig. 2 ▸), yielding values of *f*_bdy_ and Δ^*T*^_sol,bdy_, suggest greater contraction of boundary solvent than protein in all cases (by three to seven times) and a greater fraction of bulk solvent for high solvent-content crystals (Fig. 7 ▸). Using *MAP_CHANNELS*, we then calculated the boundary-layer thicknesses to be 4–8 Å, slightly increasing with larger pore sizes. This is consistent with analyses of crystal structures and molecular-dynamics simulations examining the extent of hydration water (Chen *et al.*, 2008 ▸; Bhattacharjee & Biswas, 2011 ▸). Depending on the experimental probe, water within several angstroms can be affected by the protein (Bagchi, 2013 ▸). We expect the crystal contacts to be relatively loosely organized in that surface-exposed side chains are less constrained by neighboring protein atoms, which should allow (or drive) thermal contraction greater than in the protein core. Consistent with this, solution measurements at 330 K suggest the thermal expansion of boundary solvent to be two to five times larger than for the protein (Hiebl & Maksymiw, 1991 ▸).

Combining (1) and (2) for cases of constant protein contraction Δ_prot_ (which is a good approximation at −0.013) yields two simplifications. Firstly, Δ^*T*^_sol,bdy_ = Δ^*T*^_sol,match_, or the boundary solvent contraction is identical to the solvent contraction that matches the pore contraction, consistent with the notion of the pore contraction being driven by or limited by the boundary solvent contraction. Secondly, *f*_bulk_ = (the slope of Δ_cell_ versus Δ^*T*^_sol_)/ν_sol_, or the slope normalized by the solvent content. If all of the solvent in the pore behaves as bulk, then *f*_bulk_ = 1. Table 1 ▸ shows that solvent in crystals with higher solvent content (Fig. 7 ▸) or pore sizes (Supplementary Fig. S7) have higher values of *f*_bulk_.

#### 5.1.2. Pressure-based effects   

In an alternative to the model above, the channels and the solvent contained within them have mismatched thermal contractions, which will create stress upon cooling. Temperature-induced pressurization of liquids in a solid porous matrix has been studied in geo­materials (Rice, 2006 ▸; Ghabezloo & Sulem, 2009 ▸) and is characterized by a pore pressurization coefficient Λ = (β_sol_ − β_chan_)/(κ_sol_ + κ_chan_), where β and κ are the coefficients of thermal expansion and compressibility, respectively. Here, a rough estimate suggests up to the order of ±10^1^ MPa in pore pressurization with a 100–200 K temperature decrease (Appendix *A* [App appa]).

Pore pressurization will then create (residual) strains. Tensile and compressive strains may occur, as well as shear strains in the solid phase (here the protein). Shear strains have been proposed for proteins in response to ligand binding as mechanisms for allostery (Mitchell *et al.*, 2016 ▸). Additionally, the pressure may cause flow of solvent and repacking of the proteins in the crystal lattice.

#### 5.1.3. Pressure-based effects: residual strain in the protein   

Pressure is known to compress both the protein and the unit cell. For example, at 200 MPa tetragonal lysozyme crystals show 2, 1.2 and 2.6% volume reductions of the unit cell, protein and pores, respectively (Yamada *et al.*, 2015 ▸). Figs. 1 ▸ and 2 ▸ suggest residual compressive strain in the protein on either side of Δ^*T*^_sol,match_. On either side of Δ^*T*^_sol,match_ the solvent contracts differently to the pore, which should induce pore pressurization of the fluid and exert stress on the protein with cooling. Based on RT compressibility of proteins (Appendix *A* [App appa]), the pressure required to produce the observed protein strains is up to 35 MPa. It is surprising that the protein appears to experience compression both above and below Δ^*T*^_sol,match_. However, there is some question about how to properly consider a protein crystal. It might be reasonable to model the lowest solvent-content crystals as a solid porous protein matrix with liquid in the pores. However, very high solvent-content protein crystals, the thermal response of which should be dominated by the solvent, may be more appropriately compared with a porous fluid-filled gel, which shows compression of the solid phase with cooling owing to the greater contraction of the fluid (Scherer *et al.*, 1991 ▸). Most crystals, however, have intermediate solvent contents, with possibly intermediate behavior.

#### 5.1.4. Pressure-based effects: compression from external solvent   

Compression might also happen from a mismatch in thermal contraction between the external solution and the crystal. We did not find a measurable dependence of cell volumes on the contraction of external oil, or the oil thickness, although the oils contract up to four times more than the unit cells (Supplementary Fig. S1).

#### 5.1.5. Pressure-based effects: solvent transport   

Because the solvent in the pores is liquid for at least some of the cryocooling process, there is the possibility of solvent transport along the channels owing to the pressure created by the mismatched thermal contractions. This flow is governed by the Hagen–Poiseuille equation and may be compared with solvent compression using the ratio (Appendix *B* [App appb])
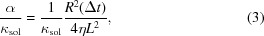
where α and κ_sol_ characterize the flow and compression responses of the channel solvent to pore pressurization, or Δ^*P*^_chan_ = (κ_sol_ + α)Δ*P*. For large values of α/κ_sol_ the response to pressure is dominated by flow, whereas for small values of α/κ_sol_ the response to pressure is dominated by compression. By setting α/κ_sol_ to 1, we have a flow-length scale on which compression and flow have similar effects. Solving (3) for *L*, and calling it *L*_crit_, yields
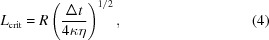
where *R* is the pore radius, Δ*t* is the cooling time and κ and η are the compressibility and viscosity of the liquid in the pore, respectively. If flow lengths are less than *L*_crit_ by a fewfold then we can ignore compression effects and most of the mis­matched contraction-caused stress will be relieved *via* flow. For pores of 10–20 Å in radius and a cooling time of 0.5 s (Teng & Moffat, 1998 ▸; Walker *et al.*, 1998 ▸; Warkentin *et al.*, 2006 ▸), we can estimate that *L*_crit_ varies from several hundred micrometres for water at room temperature to less than a micrometre for 50% xylose at 250 K (Appendix *B* [App appb]), supporting the notion of flow as a response to pore pressurization in protein crystals.

To estimate the minimal pressures required for flow, we set the compressibility *κ* = 0 and consider Δ^*T*^_sol_ − Δ_channel_ = ν_exit_ = αΔ*P* = [*R* ^2^(Δ*t*)/4η*L* ^2^]Δ*P*, which is the fraction of the solvent contained within the pore that exits the unit cell with cooling. For tetragonal thermolysin (*R* = 20 Å) ν_exit_ values are predicted to be as high as 4% (Fig. 6 ▸). Assuming that *L* = 10 µm the required pressure drop to achieve this value of ν_exit_ ranges from ∼1 kPa for water at RT to ∼4 MPa for 50% xylose at 250 K. These pressures are consistent with the residual protein strains discussed above.

For such flow to happen, there would need to be regions of low pressure in the crystal separated by a few micrometres, or more specifically distances smaller than *L*_crit_. Defect-density measurements using atomic force microscopy (AFM) suggest spacings of 3–10 µm (Malkin *et al.*, 1996 ▸; Dobrianov *et al.*, 1999 ▸; Kriminski *et al.*, 2002 ▸). Superfine φ-slicing measurements of cryocooled β-galactosidase crystals suggest domain sizes of ∼0.5–10 µm (Juers *et al.*, 2007 ▸). Tetragonal thermolysin often grows out of precipitate, and precipitate-like material can be seen visually within the crystals. Further, new defects may be created during cooling *via* disruption of the crystal by the pore pressurization (Malkin & Thorne, 2004 ▸). Crystal defects and inclusions might therefore provide regions of low pressure that act as sinks for extruded solvent.

If *L*_crit_ is larger than the crystal then the extra solvent would be completely removed from the crystal. This could occur for very small crystals, with very low viscosity cryosolvents or for slowly cooled crystals. At the other extreme very small values of *L*_crit_ (for example a few angstroms) would result in very little flow, with most of the stress being relieved by solvent compression. Because the relevant physical parameters (β, κ and η) are temperature-dependent, a crystal may go from one extreme to the other during cooling. A more detailed view may therefore emerge from studying the behavior of these systems between RT and LT.

In summary, a combination of modulation of solvent properties by pore confinement, as well as pressure-induced compression of proteins and flow of solvent, can explain the overall mismatch between channel contraction, Δ_chan_, and solvent contraction, Δ^*T*^_sol_, and the smaller dependence of Δ_chan_ on Δ^*T*^_sol_ for low solvent-content crystals. We turn next to the negative slope of Δ_chan_ *versus* Δ^*T*^_sol_ for some expanding solvents.

#### 5.1.6. Diffusion-based transport mechanisms and ice formation   

One surprising result was the reduction of cell and pore volumes at low cryoprotectant concentrations (Figs. 2 ▸ and 6 ▸). Orthorhombic trypsin showed up to an 8% additional pore contraction when switching from 30 to 20% xylose, even though the latter solution expands more with cooling. This greater contraction would require about 160 MPa of pressure to be owing to compression (using the compressibility of water at 273 K of ∼50 × 10^−6^ atm^−1^; Yamada *et al.*, 2015 ▸), but we actually observe the smallest protein contraction under these conditions (Fig. 2 ▸), suggesting that the protein is under tension relative to Δ^*T*^_sol,match_. This greater channel contraction may alternatively be explained by mass transport to feed ice-crystal growth, which probably initiates in voids within the crystal or on the outside of the crystal. As these ice crystals start to grow, the cryosolvent will still be liquid in the narrower pores (owing to the Gibbs–Thomson effect), allowing diffusion. The growing ice crystal will create solute-rich regions in its adjacent liquid phase, and therefore a chemical potential favoring the diffusion of water towards the ice crystal. Similar mechanisms of ‘solvent draining’ have been suggested for the unit-cell shrinkage concomitant with ice formation during the warming of flash-cooled trypsin crystals (Weik *et al.*, 2005 ▸), as well as the cooling-induced contraction of concrete prepared with air pockets which serve as reservoirs for ice-crystal growth (Sun & Scherer, 2010 ▸). Impressively, Supplementary Fig. S8 suggests that even with hexagonal ice formation this process can be largely reversible in trypsin crystals.

Cryoprotective agents both reduce the freezing point of ice (*T*_c_) and in some cases increase the glass-transition temperature (*T*_g_) (Shah & Schall, 2006 ▸). Thus, for lower cryoprotective agent concentrations the onset of ice crystallization would happen at a higher temperature, when the viscosity of the cryosolution is lower, and last a longer time since the *T*_c_–*T*_g_ gap is larger. More water would diffuse out, reducing the cell volume more (Fig. 2 ▸). Smaller solvent channels may not support this mechanism since the viscosity of confined water is strongly dependent on pore size below radii of ∼7 Å (*i.e.* 10^3^–10^6^ times the bulk value for pore radii of 5 Å; Wu *et al.*, 2017 ▸). With larger solvent channels, some ice may form inside the channels, causing an extra expansion of the cell and channel, as is observed for tetragonal thermolysin at Δ^*T*^_sol_ = 1% (Fig. 6 ▸). This mechanism is similar to events during slow-cooling approaches to the cryopreservation of tissues, in which ice formation is allowed to occur outside sensitive regions such as cells, which draws water out of the cells, shrinking them and increasing the solute concentration inside (Pegg, 2007 ▸).

### 5.2. Response of protein conformation and crystal packing to cooling and cryosolvent contraction   

In addition to modulation of the overall protein contraction, we see variation in protein conformation with Δ^*T*^_sol_. Thermolysin hinge-bending angles depend systematically on solvent contraction (Fig. 3 ▸ *a*). Protein conformational differences between LT crystals with different cryosolvents can be as great as the differences observed between RT and LT crystals (Fig. 3 ▸ *b*). At the same time, LT conformational differences can be very small if the cryosolvents have similar contractions. Therefore, to understand binding effects using cryocrystallography it is crucial that the native enzyme and the ligand-bound enzyme be cooled identically, so that the conformational differences reflect ligand binding and not coupling to solvents with different contractile tendencies. Alternatively, different cryosolvents may be used to explore conformational variation for a particular protein.

Packing density at intermolecular contacts is significantly impacted by the cryosolvent contraction. The highest contracting solvents are correlated with the greatest number of crystal contacts at LT, suggesting that these solvents pull the protein molecules more tightly together in the crystal lattice (or alternatively that the intrinsic tendency of the protein molecules to become closer together is permitted by the high solvent contraction).

### 5.3. The effects of cryocooling and cryosolution contraction on crystal order   

When a crystal is cryocooled, often the mosaicity increases and the diffraction power is reduced. Here, it is clear for some crystals that the LT mosaicity can be minimized by screening the cryosolution thermal contraction.

The exact nature of the cooling-induced disorder is not very well understood. At its most basic level, the mosaicity increase is owing to different unit cells responding differently to the cooling process, producing variation in cell dimensions and orientations throughout the crystal (Darwin, 1922 ▸). Mosaicity changes have been modeled using domains, with increased angular spread between domains, unit-cell variation between or within domains and reduced domain sizes contributing to increases in mosaicity with cooling (Nave, 1998 ▸; Kriminski *et al.*, 2002 ▸; Juers *et al.*, 2007 ▸; Vahedi-Faridi *et al.*, 2003 ▸). We note that within this model a 1° increase in mosaicity at 3 Å resolution corresponds to a 1° angular deviation between domains, a 1.7% variation in unit-cell length or a domain size of ∼200 nm.

Cooling-induced damage can happen *via* inhomogeneous and homogeneous processes (Kriminski *et al.*, 2003 ▸). The former arise from temperature gradients, which cause differences in cell volumes over small distances. If the resulting strain is too high the system deforms plastically, increasing the mosaicity. Inhomogeneous processes should be more significant for larger cell contractions (*i.e.* equation 14 of Kriminski *et al.*, 2003 ▸), which correlates with our observations that greater cell contractions produce greater mosaicities. However, cooling slowly, such as *via* gas-stream cooling as employed here, is expected to limit inhomogeneous damage (Kriminski *et al.*, 2003 ▸).

When cooled homogeneously, crystals can suffer damage presumably because different parts of the crystal are responding differently to the uniform temperature change. Even crystals cooled at 0.1 K s^−1^ in the absence of ice formation show mosaicity increases, most likely through homogeneous processes (Warkentin & Thorne, 2009 ▸), which could include the transport of solvent out of (or in to) unit cells as discussed above. This would increase the angular spread between unit cells or domains, and variation in the amount of solvent flowing should create unit-cell variation throughout the crystal. Greater pressure will increase ν_exit_, increasing the angular spread between domains. Greater pressure might also create defects for pooling extruded solvent, reducing the defect spacing and therefore the domain size. This compounding of effects with increased pressure could explain the nonlinearity of mosaicity *versus* Δ^*T*^_sol_ (Fig. 4 ▸ *a* and Supplementary Fig. S4).

It should be noted that multiple physical characterisics of cryosolvents change with the thermal contraction. For example, MeOH is one of the largest contractors (13% *versus* 4% for glycerol), but it also has a very low melting point (175 K *versus* 290 K for glycerol) and viscosity, so we may expect solutions of methanol to flow more easily down to a lower temperature than some other cryoprotective agents.

Cooling that causes conformational rearrangement and repacking of the crystal lattice may increase disorder unless the free-energy landscape points to a very clearly defined new minimum and the cooling rate is slow enough to permit the entire crystal to transition to this state. Otherwise the crystal system will become quenched in a range of packing arrangements. Similar arguments have been made for conformational variability in the proteins themselves (Halle, 2004 ▸).

For the crystals tested here, the contraction of an external oil had no measurable systematic effects on mosaicity. This is probably related to the fact that although sometimes the external oil contracts more than the crystal inside it, while this contraction is happening the whole system is unconstrained and the external cryosolvent can flow to accommodate the relatively incompressible material beneath. It is possible that external aqueous cryosolutions could impact crystal order, especially for fragile rods and plates, but probably from crystal warping rather than uniform compression from contraction of the external solvent. With the exception of the rod-shaped hexagonal thermolysin, the crystals tested here were relatively chunky, with appreciable thicknesses in all three dimensions, and were therefore probably resistant to warping. For rods and plates, crystals can be mounted using specialized loops designed for this purpose. Additionally, using surface tension only (*i.e.* a loop larger than the crystal) can improve LT mosaicities for such crystals.

A mosaic block model predicts that 4% of the solvent exiting the unit cell owing to mismatched Δ_chan_ and Δ^*T*^_sol_ would cause a maximum angular deviation of mosaic blocks of about 0.5° (Appendix *C* [App appc]). This value compares favorably with the mosaicity increases observed for tetragonal thermolysin. However, this simple model does not account for reduction in domain sizes or unit-cell variation. Lower solvent-content crystals have larger mismatches between Δ_chan_ and Δ^*T*^_sol_, but the smaller pore size probably suppresses the bulk behavior of the solvent in these crystals to a greater degree.

### 5.4. Cryoprotection optimization   

Our results point to some general principles to be considered in optimizing cryoprotection by considering the contraction of the cryosolvent and the solvent content of the crystal.

#### 5.4.1. Thermal-contraction-guided cryo-optimization   

Some of the crystals tested here show a correlation between crystal order and cryosolvent thermal contraction. For some crystals, it will therefore be helpful to systematically vary the thermal contraction of the cryosolutions during cryo-optimization, as has been demonstrated with α-lactalbumin. It seems reasonable to first try a low-contracting cryosolvent, perhaps about 3% contraction.

While it is clear that solvent thermal contraction can be anticorrelated with crystal order, it is less clear whether there is an optimal solvent contraction value matching the pore contraction (for example 5% for tetragonal thermolysin) or crystal contraction. In most cases an optimum is not evident. Two cases hint at an optimum which corresponds to cell contractions of 3–4% for α-lactalbumin and 4–5% for hexagonal thermolysin. Tetragonal thermolysin shows the lowest mosaicity and *B* factor at the lowest contraction achievable without ice formation, corresponding to a cell contraction of 0–3%. Faster cooling methods to prevent ice formation may shed more light on the behavior of tetragonal thermolysin, although this may also increase inhomogeneous dis­ordering (Kriminski *et al.*, 2003 ▸).

For hexagonal thermolysin, reduction of cryoprotectant concentrations to match the protein contraction (∼1%) appears to create more stress with cooling. For example, elimination of ice formation in hexagonal thermolysin using oils still produced crystals with higher mosaicities with low-contracting internal cryoprotectants (*i.e.* 0–20% xylose; Supplementary Fig. S5). There appears, therefore, to be a mismatch between the instrinsic contractions of the protein (determined by core packing effects) and the pore (determined by both core packing and surface effects), which cannot be avoided. Hence, there will be some amount of stress created even when the cryosolvent is optimized. More experiments in this area are, however, warranted.

Previous studies have indicated cyosolution optima. For example, Mitchell and Garman studied crystals of glycogen phosphorylase B (50% solvent, maximum pore radius 7.2 Å), showing an optimum glycerol concentration for cryocooling of about 45%(*w*/*v*) (Mitchell & Garman, 1994 ▸) corresponding to a solvent contraction of about 4.5% (Shen *et al.*, 2016 ▸), which falls in the above range.

It is unclear what governs the steepness of the mosaicity decreases with Δ^*T*^_sol_ in Fig. 4 ▸(*a*) and Supplementary Fig. S5. More rapid cooling should have a larger effect on the higher contractors owing to inhomogeneous effects, increasing the steepness. The tolerance of crystals to a range of thermal contractions appears to involve structural elasticity within and between proteins in the crystal. Steeper mosaicity decreases are therefore likely for crystals with very weak contacts relative to internal rigidity (*i.e.* fragile crystals).

We note that at room temperature the mosaicity is dominated by the beam divergence (5.2 mrad or 0.3°) and wavelength spread of our X-ray source. Cooling is known to increase the true crystal mosaicity severalfold (Vahedi-Faridi *et al.*, 2003 ▸). However, for some of the cryosolutions tested here the mosaicity is still dominated by beam characteristics even at low temperature. More information may thus be uncovered by using a synchrotron-radiation source, especially without focusing optics, in which case mosaicity changes with cooling will represent a larger fraction of the base mosaicity (Bellamy *et al.*, 2000 ▸)

Cryo-optimization processes can then include knowledge about the cryosolvent thermal contraction. Information about the effects of the cryoprotective agent as well as concentration on contraction are available (Juers & Matthews, 2004*a* ▸; Alcorn & Juers, 2010 ▸; Shen *et al.*, 2016 ▸, 2017 ▸). From these studies, some general principles are known. More hydrophobic solutions tend to contract more. In typical ranges of cryoprotective agent concentrations, the contractions vary a fair amount. The range of 3–5% corresponds to about 35–45%(*w*/*w*) glycerol, 30–40%(*w*/*w*) ethylene glycol and 25–30%(*w*/*w*) MPD. The impacts of other solutes on these contractions has not yet been reported, nor has information about the continuous thermal contraction between 300 and 200 K, which is the range over which damage is likely to occur, as can be seen in Fig. 4 ▸ of Warkentin & Thorne (2009 ▸).

#### 5.4.2. Crystal solvent content as a guiding parameter   

The results here demonstrate that the thermal behavior of a protein crystal is strongly dependent on the pore size or the solvent content. Over the range of common protein crystal pore sizes, the freezing point of water in porous silica varies by more than 100 K. Our results suggest that for pore radii smaller than a few angstroms the bulk-solvent contraction will have essentially no effect on cell volumes or mosaicities. This means that both high contractors and low contractors can be explored. High-quality diffraction even with ice formation is probable. Elimination of ice rings by using external oils, by wicking or using any aqueous cryosolution are likely to give positive results.

We calculated pore sizes for a nonredundant subset of structures in the Protein Data Bank (about 45 000 structures) and found the mean maximum pore radius to be about 12 Å. Therefore, more than half of protein crystals should have a thermal response modulated by solvent contraction (Fig. S7). For these crystals, adjusting the solvent contraction may reduce low-temperature mosaicity and keeping the solvent contraction constant will improve isomorphism between crystals.

Depending on the composition of the solvent, some of these crystals will only require the removal of external solution. For example, Warkentin and Thorne showed that thaumatin crystals grown from 1.5 *M* sodium potassium tartrate could be both flash-cooled and slow-cooled simply by using NVH oil and carefully removing all aqueous external solvent (Warkentin & Thorne, 2009 ▸). Our results suggest that the presence of the salt is important to give the solvent ample contraction. The cell contraction in this case was about 4%, corresponding to a solvent contraction of about 7% (Fig. 2 ▸). Pellegrini and coworkers showed that several crystals (solvent content 44–47%) could be successfully flash-cooled simply by wicking away all external solution (Pellegrini *et al.*, 2011 ▸), an approach that has been adopted for automounting of crystals (Zander *et al.*, 2016 ▸). Our results suggest that some of these crystals may still benefit from solvent-contraction optimization.

Other crystals, especially high solvent-content crystals with large pores, will require penetrating cosolvents to achieve the correct solvent contraction. These types of crystals are the most likely to benefit from solvent-contraction optimization. We suggest starting with a cryosolvent that contracts by ∼3% and then systematically adjusting the cryosolvent and its contraction to find optimal diffraction conditions.

## 6. Conclusions   

Our results demonstrate that solvent thermal contraction can impact the thermal response of macromolecular crystals and proteins. The effects appear to be both directly from the temperature change and also from pressure caused by mismatched solvent and pore thermal contraction. Damage from ice formation can include a reduction of cell volumes *via* what appears to be transport of liquid along solvent channels during cooling. In some cases, adjustment of solution thermal contraction can be used to limit cooling-induced crystal damage.

## Supplementary Material

PDB reference: tetragonal thermolysin, 5un3

PDB reference: 5uu7

PDB reference: 5uu8

PDB reference: 5uu9

PDB reference: 5uua

PDB reference: 5uub

PDB reference: 5uuc

PDB reference: 5uud

PDB reference: 5uue

PDB reference: orthorhombic trypsin, 6avl

PDB reference: 6b6n

PDB reference: 6b6o

PDB reference: 6b6p

PDB reference: 6b6q

PDB reference: 6b6r

PDB reference: 6b6s

PDB reference: 6b6t

PDB reference: 6dzf

PDB reference: triclinic lysozyme, 6d6e

PDB reference: 6d6f

PDB reference: 6d6g

PDB reference: 6d6h

PDB reference: hexagonal thermolysin, 6d5n

PDB reference: 6d5o

PDB reference: 6d5p

PDB reference: 6d5q

PDB reference: 6d5r

PDB reference: 6d5s

PDB reference: 6d5t

PDB reference: 6d5u

Additional figures, data-collection and refinement statistics and information about solution thermal contractions.. DOI: 10.1107/S2059798318008793/tz5096sup1.pdf

Crystal data.. DOI: 10.1107/S2059798318008793/tz5096sup2.xls

## Figures and Tables

**Figure 8:**
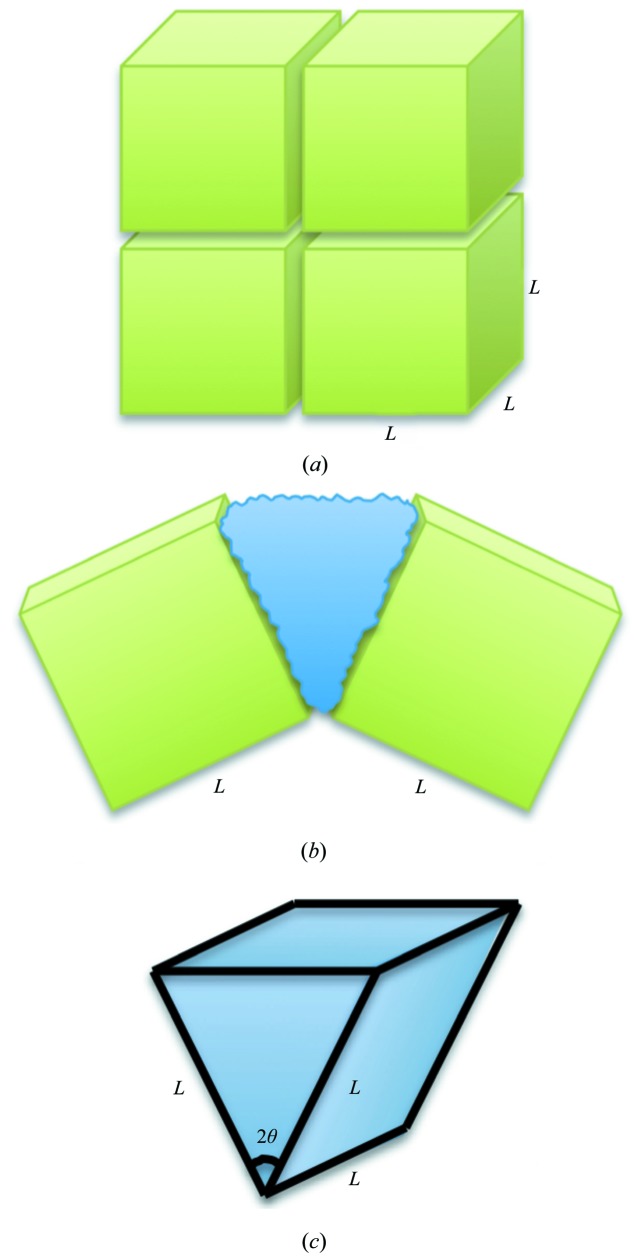
Schematics showing the effect of solution extruded from a crystal during cooling on mosaicity. (*a*) Representation of four domains in a protein crystal, at room temperature, each of length, width and height *L*. (*b*) Representation of two crystal domains during cooling: the solvent (blue) flows a length *L* and is extruded into the space between the domains, displacing each domain by some angle. (*c*) Geometric representation of solvent extruded between domains during cooling. Each domain is displaced by the exiting solvent by an angle θ, giving an angle of 2θ for the volume of solution extruded between two crystal domains of length *L*.

**Table 1:** Calculated properties of the protein crystals investigated The RT solvent content, ν_sol_, is based on a *V*_M_ calculation (Matthews, 1968 ▸). The maximum pore radius (Å) was calculated with *MAP_CHANNELS* (Juers & Ruffin, 2014 ▸) using the coordinates from RT xylose soaks determined here (triclinic lysozyme, orthorhombic trypsin, hexagonal thermolysin and tetragonal thermolysin) and, for the other proteins, structures in the Protein Data Bank with similar cell dimensions to the RT crystals used here (trigonal trypsin, PDB entry 1ghz; tetragonal lysozyme, PDB entry 5kxo; thaumatin, PDB entry 5kw3; insulin, PDB entry 1b2g; Katz *et al.*, 2001 ▸; Russi *et al.*, 2017 ▸; Diao, 2003 ▸). The boundary solvent contraction and bulk fraction, Δ^*T*^_sol,bdy_ and *f*_bulk_, are from fits of (1) and (2) to data in Fig. 2 ▸. For constant Δ_prot_, *f*_bulk_ is the normalized slope (by the solvent content) of Δ_cell_ *versus* Δ^*T*^_sol_ and Δ^*T*^_sol,bdy_ = Δ^*T*^_sol,match_, the value of Δ^*T*^_sol_ that matches the pore contraction. The maximum pore radius is the radius of the largest spherical object which could fit inside, but not necessarily transit, the solvent channels.

Crystal	ν_sol_	Maximum pore radius (Å)	Δ^*T*^_sol,bdy_ = Δ^*T*^_sol,match_	*f*_bulk_
Triclinic lysozyme	0.32	5.5	−0.085	0.10
Trigonal trypsin	0.40	6.1	−0.048	0.19
Tetragonal lysozyme	0.41	7.7	−0.066	0.32
Orthorhombic trypsin	0.46	10.1	−0.056	0.21
Hexagonal thermolysin	0.49	13.1	−0.077	0.38
Thaumatin	0.58	12.9	−0.051	0.39
Insulin	0.65	17.4	−0.045	0.52
Tetragonal thermolysin	0.67	20.0	−0.049	0.41

## Appendix A Abbreviations, symbols and viscoelastic parameters

The thermal response of protein crystals depends on intrinsic material thermoelastic properties: the thermal expansivity [β ≡ (1/*V*)(∂*V*/∂*T*)_*P*_], the compressibility [κ ≡ (−1/*V*)(∂*V*/∂*P*)_*T*_] and the viscosity (η). Here, we summarize information about these properties for the crystal components.

### A1. β: thermal expansivity (〈 〉 indicates an average over the specified temperature range)

〈β_sol_〉: 5.9 × 10^−4^ K^−1^ (50% MeOH; 295–77 K; Alcorn & Juers, 2010 ▸); 3.9 × 10^−4^ K^−1^ (50% MPD; 295–77 K; Alcorn & Juers, 2010 ▸); 1.4 × 10^−4^ K^−1^ (50% xylose; 295–77 K; Alcorn & Juers, 2010 ▸); ∼−1.4 × 10^−4^ K^−1^ (30% xylose; 295–77 K; this work).
〈β_cell_〉: 1–3 × 10^−4^ K^−1^ (various; 2–6% contraction for 295–100 K; this work; Frauenfelder *et al.*, 1987 ▸; Juers & Matthews, 2001 ▸; Fraser *et al.*, 2011 ▸).
〈β_prot_〉: 0.7–0.9 × 10^−4^ K^−1^(various; 1.3–1.8% contraction for 295–100 K; this work).
〈β_chan_〉: 2.6 × 10^−4^ K^−1^ (for tetragonal thermolysin, 5.5% contraction for 295–100 K; this work, estimated as Δ^*T*^_sol,match_).
Confinement effects: MD simulations suggest an increase in the thermal expansion of water near the boundary layer of porous silicon by twofold to fourfold at 293 K (Xu *et al.*, 2009 ▸).

### A2. κ: compressibility (〈 〉 indicates an average over the specified temperature range)

〈κ_sol_〉: 0.5–0.8 GPa^−1^ (supercooled water; 300–240 K); 0.25 GPa^−1^ (50% glucose; Randall, 1931 ▸).
〈κ_cell_〉: 0.10 GPa^−1^ (lysozyme; 2% contraction for 200 MPa; Yamada *et al.*, 2015 ▸).
〈κ_prot_〉: 0.06 GPa^−1^ (lysozyme; 1.2% contraction for 200 MPa; Yamada *et al.*, 2015 ▸).
〈κ_chan_〉: 0.13 GPa^−1^ (lysozyme; 2.6% contraction for 200 MPa; Yamada *et al.*, 2015 ▸).
Confinement effects: there is little information about the compressibility of confined water. Confined argon has been shown *via* MD to have lower compressibility in smaller pores (Gor *et al.*, 2015 ▸).

### A3. η: viscosity

Supercooled water: 1–20 mPa s (300–240 K; Dehaoui *et al.*, 2015 ▸).
50% ethylene glycol: 5–100 mPa s (300–240 K; Dortmund Data Bank, http://ddbonline.ddbst.de/).
Methanol, 0.5–1.6 mPa s (300–240 K; Dortmund Data Bank, http://ddbonline.ddbst.de/).
50% glucose, 10 mPa s (RT; Soesanto & Williams, 1981 ▸).
Confinement effects: confinement of water to hydrophilic pores greater than 7 Å in radius increases the viscosity by twofold to threefold over its bulk value at RT (Wu *et al.*, 2017 ▸). For smaller pores the viscosity of water is 10^3^–10^6^ higher. We can therefore suppose the viscosity of the cryoprotective agents in the pores to be at least 10^2^–10^3^ mPa s; however, this is quite uncertain and may vary substantially amongst the protein crystals investigated.

The pore pressurization coefficient Λ = (β_sol_ − β_chan_)/(κ_sol_ + κ_chan_) (Rice, 2006 ▸; Ghabezloo & Sulem, 2009 ▸).

Using the above values of β_sol_, β_chan_, κ_sol_ and κ_chan_ gives values of Λ up to ±0.5 MPa K^−1^ or of the order of ±10^1^ MPa for Δ*T* = 100–200 K. However, the appropriate value of κ_chan_ would be that for constant external pressure while varying the pore pressure, while the experiment of Yamada *et al.* (2015 ▸) involved an isotropic external stress applied to the whole protein crystal. Keeping the external pressure constant would increase κ_chan_, reducing Λ. Thus, the values above may be considered to be an upper limit for temperature-induced change in pore pressure.

## Appendix B Flow response to pressure

A pressure increase with cooling owing to thermal expansion mismatch, Δ*P* ≅ ΔβΔ*T*/(κ_chan_ + κ_sol_), may extrude solvent out of the unit cell to voids or outside the crystal where the pressure is lower, allowing a pore pressure gradient along the pore. We model this flow using the Hagen–Poiseuille equation for laminar flow in a pipe,

where 〈*v*〉 is the average velocity of the fluid, Δ*P* is the pressure drop along the channel giving rise to the flow of the fluid, *R* is the radius of the channel, η is the viscosity of the fluid and *L* is the length of the channel. The total flow out of one end of the channel can be defined as the movement of the fluid from the midpoint to the end of the channel. Because the channels in the protein crystal are open at both ends, only half of the length of the channel needs to be considered for the total flow, *Q*_end_, which is described as volume per time:

Δ*t* is the timescale for the flow and ν_exit_ is the volume fraction of the solvent in the channel exiting during the flow. *Q*_end_ is related to 〈*v*〉, yielding

Solving for *v*_exit_ yields

We now define α = *R* ^2^(Δ*t*)/4η*L* ^2^, which characterizes the flow response of the solvent owing to pressure. The solvent-volume change owing to pressure is then given by

Here, a useful quantity to consider is

which when set to 1 allows us to define a length scale for which compression and flow have similar effects on Δ_chan_,

### b1. Verifying laminar flow

Hagen–Poiseuille flow requires Reynold numbers of less than 1,

where ρ_f_ is the density of the fluid, *v* is the velocity of the fluid, *R* is the radius of the pipe and  is the viscosity of the fluid. Evaluating the Reynolds number using the values ρ_f_ = 1.1 kg l^−1^ for a 50%(*w*/*w*) glucose solution, *v* = 2 × 10^−5^ m s^−1^ [an upper limit obtained from using (7) with ν_exit_ = 0.10, *L* as the length of the crystal (400 µm) and Δ*t* approximated as 0.5 s for the cooling time of the crystal; Teng & Moffat, 1998 ▸], *R* = 21.0 Å (a large channel radius for a protein crystal, *i.e.* tetragonal thermolysin) and η = 0.01–1 Pa s (the range of expected viscosities of pore solutions; Appendix *A*) gives Reynolds numbers of 10^−14^–10^−17^, which indicates that the cryoprotectant in the protein-crystal situation obeys the laws of laminar flow. Thus, the Hagen–Poiseuille equation is appropriate to use in (5).

The calculated Reynolds numbers are very low, indicating that any changes in the crystal that occur as a result of flow are highly reversible. This property might have implications in contexts such as the annealing of protein crystals, when the reheating of a crystal might cause a reverse in the flow. If this is true, then it would be expected that the initial damage caused by laminarly moving liquid in the crystal would be reversed when the crystal is heated. Indeed, an increase in data quality and a decrease in mosaicity have been observed using some annealing techniques (Harp *et al.*, 1998 ▸; Yeh & Hol, 1998 ▸).

## Appendix C Mosaicity owing to increased angular spread from solvent extrusion

When the solvent is extruded from the channel, the domains of the crystal will be forced apart and rotate by some amount, creating an increase in crystal mosaicity. We assume that the crystal domain has a length, width and height of *L*, and solvent flows in each direction. Each domain then has a volume (*V*_domain_) of *L* ^3^. The volume extruded from each domain can be written as

(α·Δ*P*) = ν_exit_ is the amount of flow occurring in the crystal during cooling (assuming no solvent compression). As an upper limit estimate (assuming no solvent compression and complete bulk behavior of the solvent in the channel), ν_exit_ = Δ^*T*^_sol_ − Δ_chan_. In (13), the volume extruded by one domain is divided by six, as there are six faces to each domain, and multiplied by two, as this equation considers the volume of solution extruded by two domains.

The volume of fluid extruded can also be characterized geometrically as a triangular prism, as shown in Fig. 8 ▸,

Then, (13) and (14) together yield

where θ will be equal to the portion of mosaicity that the crystal gains during cooling owing to the flow of solvent owing to angular spread between domains.

Evaluating (15) with ν_exit_ = 0.67 and Δ^*T*^_sol_ − Δ_chan_ = 0.04, which is the largest mismatch between Δ_chan_ and Δ^*T*^_sol_ for tetragonal thermolysin, results in θ = 0.5°.
