# Supplementary material for: The impact of cryosolution thermal contraction on proteins and protein crystals: volumes, conformation and order
Source: Acta Crystallogr D Struct Biol. 2018 Sep 5;74(Pt 9):922–38. doi: 10.1107/S2059798318008793 (PMC6130464; doi:10.1107/S2059798318008793)
Supplement: Supplementary file 1 [file d-74-00922-sup1.pdf]

STRUCTURAL BIOLOGY

Volume 74 (2018)

Supporting information for article:

The impact of cryosolution thermal contraction on proteins and protein crystals: volumes, conformation and order

Douglas H. Juers, Christopher A. Farley, Christopher P. Saxby, Rosemary A. Cotter, Jackson K. B. Cahn, R. Conor Holton-Burke, Kaitlin Harrison and Zhenguo Wu

Supporting information

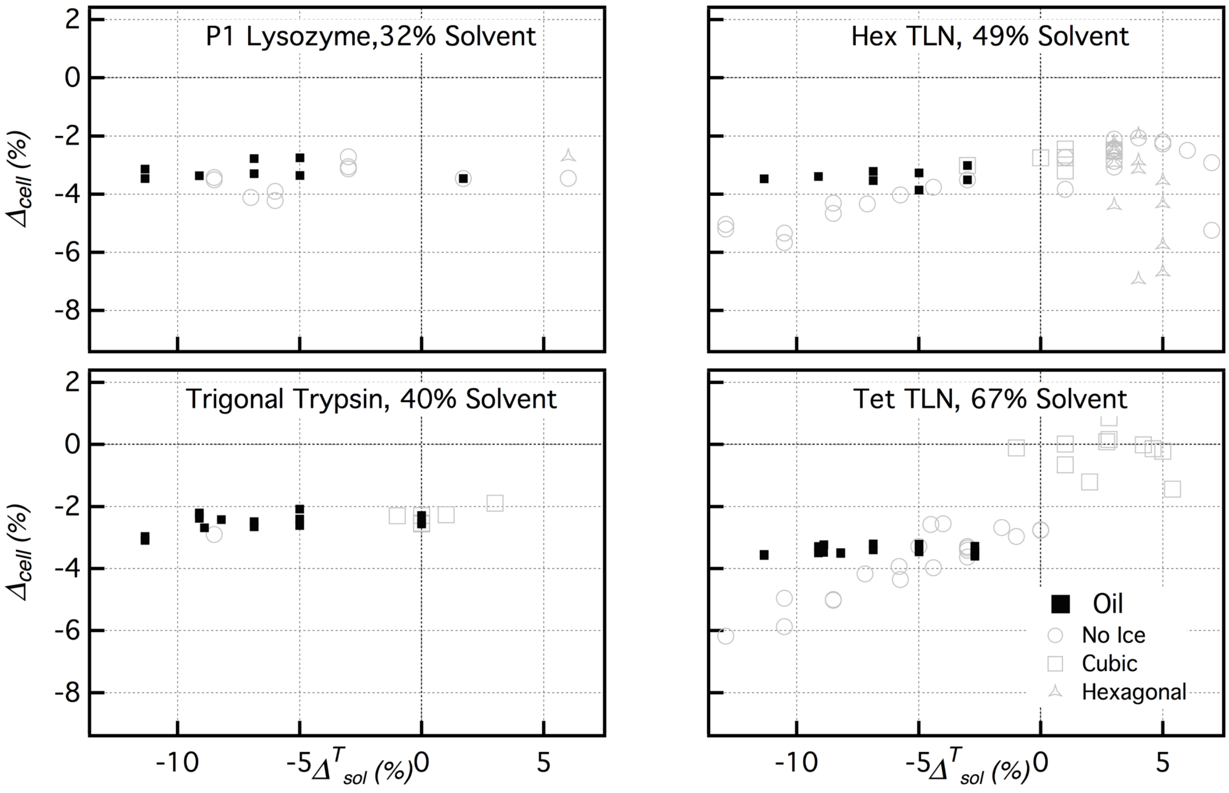

Figure S1 (a) The effects of external oil on cell volume contraction. This plot shows data from two types of experiments. For grey data points, crystals were equilibrated to an aqueous penetrating cryosolution and then flash cooled – these are identical to data from Fig 4a; Δ^T_{sol} refers to the contraction of the aqueous cryosolution. For the black square data points, crystals were first equilibrated to an aqueous solution (internal cryoprotectant) and then in most cases coated in an oil (see Methods); Δ^T_{sol} refers to the contraction of the oil, which ranged from 11.3% (paraffin oil) to 5.0% (Fomblin YR1800). Black square data points to the right of the Fomblin contraction values are negative controls in which the oil was not used, and the external aqueous solution was retained; in these cases Δ^T_{sol} refers to the contraction of the aqueous internal/external cryosolution. The internal cryoprotectants used were: triclinic lysozyme (23.5% xylose), trigonal trypsin (well solution), hexagonal thermolysin (50% xylose), tetragonal thermolysin (50% glucose).

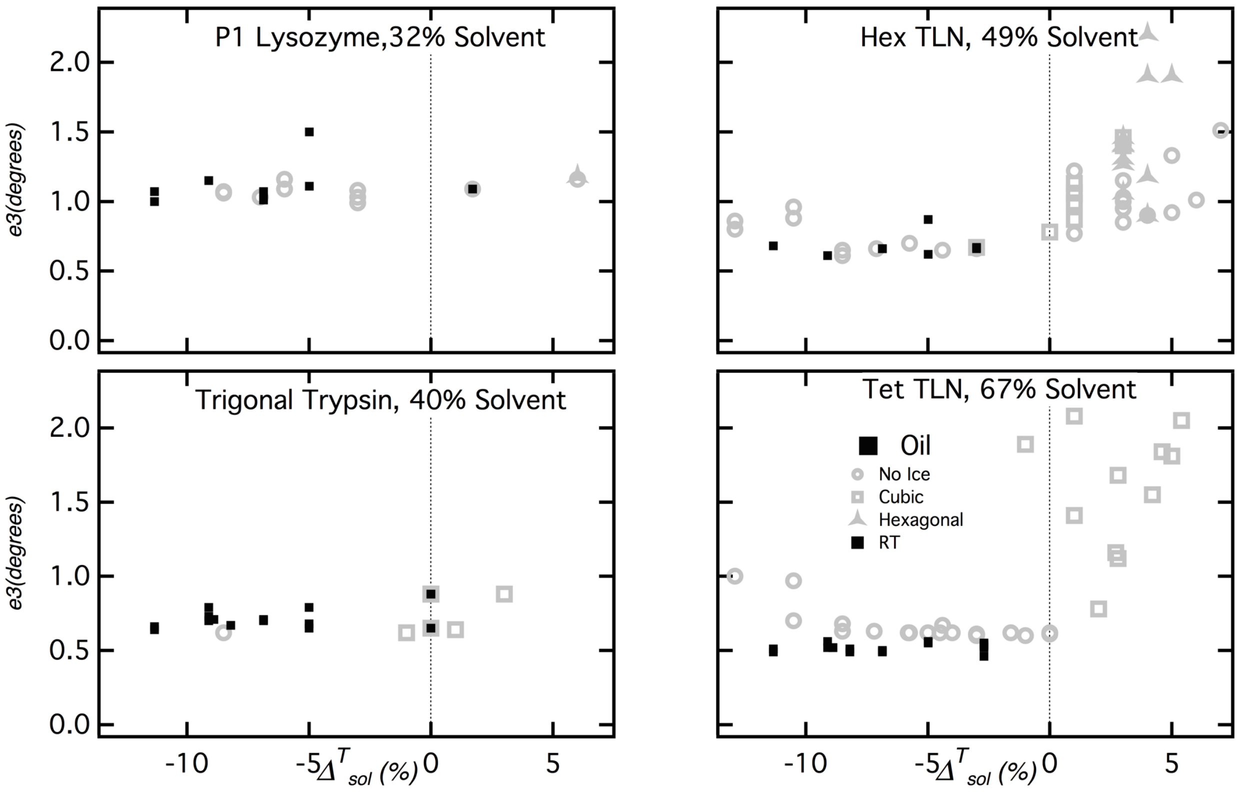

(b) Identical to Fig S1(a), but plotting the LT e3 mosaicity rather than Δ_{cell}.

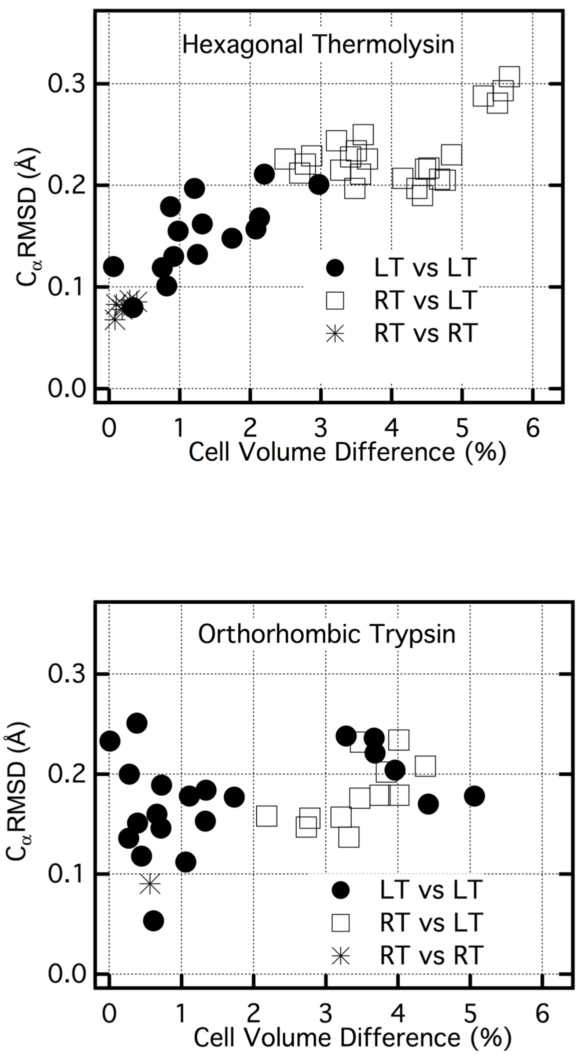

Figure S2 Dependence of structural difference (C_α RMSD) on unit cell difference for hexagonal thermolysin (a) and orthorhombic trypsin (b). Both are qualitatively similar to tetragonal thermolysin (Fig 3b)

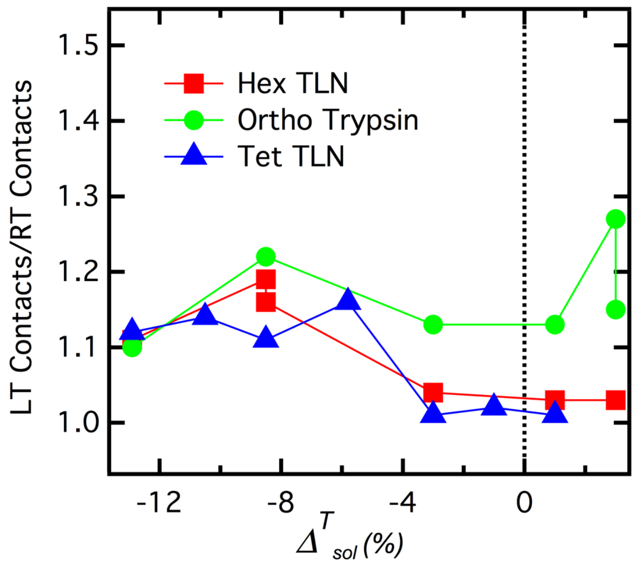

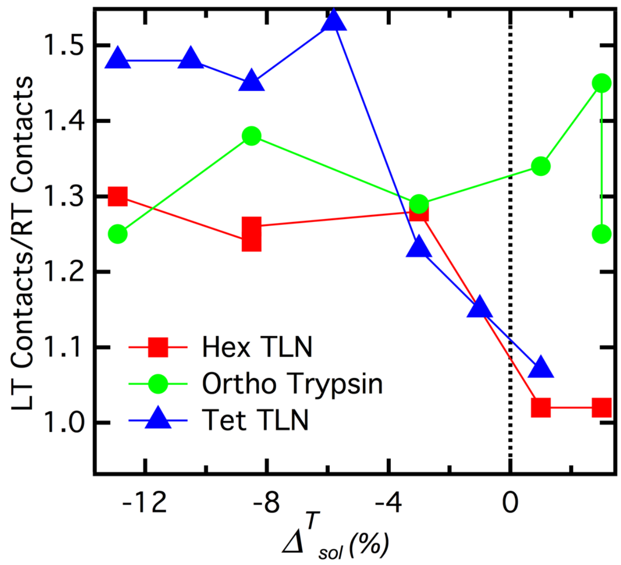

Figure S3 Crystal contact ratios similar to Fig 3c, but using different levels of stringency to calculate contacts. (Top) Contacts were calculated using a uniform 4.5 Å center-to-center cutoff distance for all atoms (Frauenfelder *et al.*, 1987). (Bottom) Contacts were calculated as all atoms pairs within 0.25 Å of the sum of van der Waals radii (Juers & Matthews, 2001) using radii as defined in Table VI of Li & Nussinov (Li & Nussinov, 1998). This more stringent definition yields 3-5x fewer contacts for all proteins, but a greater fractional increase with cooling. LT structures with positive values of Δ^T_{sol} showed some ice formation. Note the highest ratio for trypsin occurred with the greatest cell reduction at 20% xylose with ice formation.

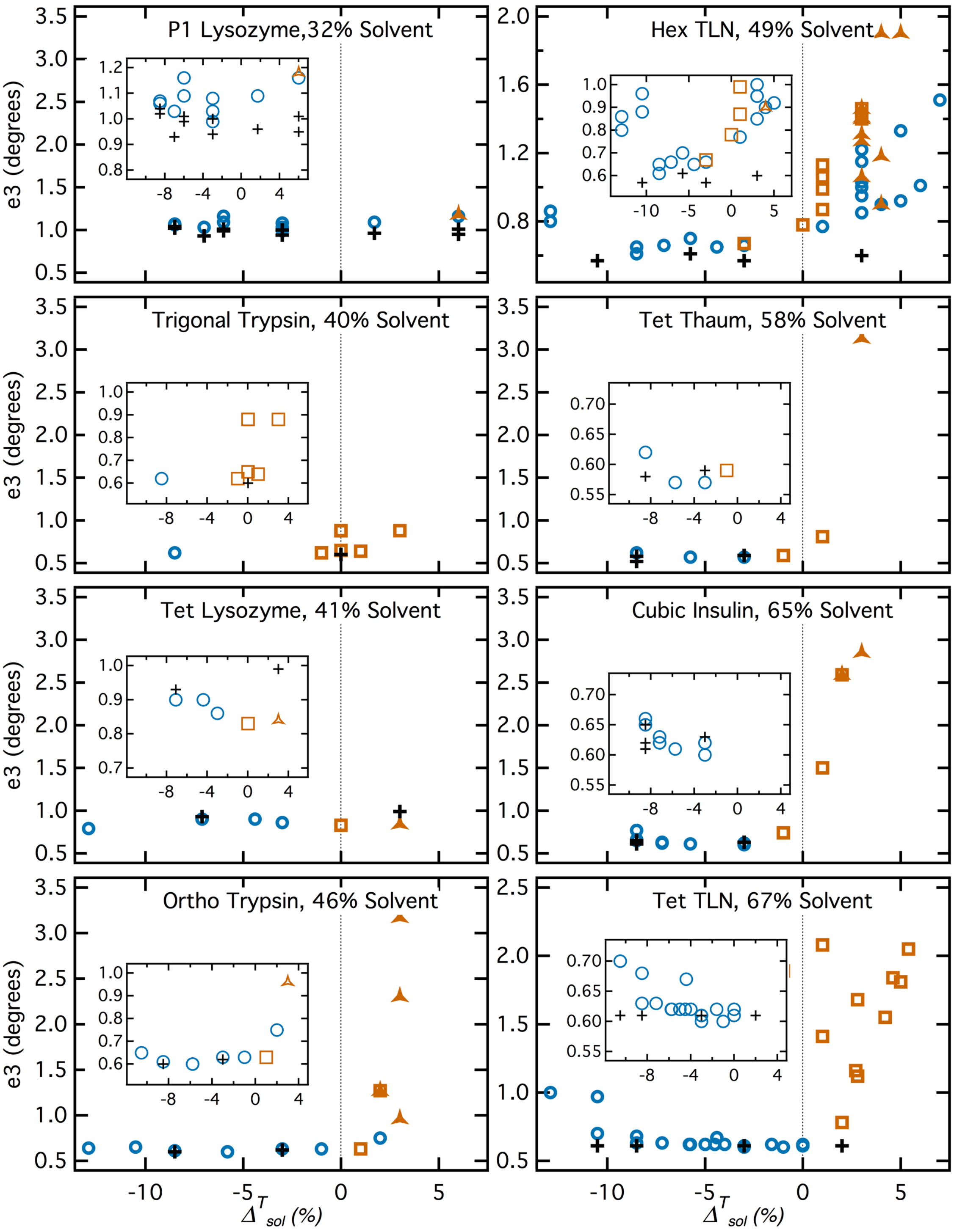

Figure S4 e3 mosaicity vs solvent contraction for all 8 crystal tested. Blue circles = LT, no ice; red squares = LT, cubic ice; red stars = LT, hexagonal ice; crosses = RT

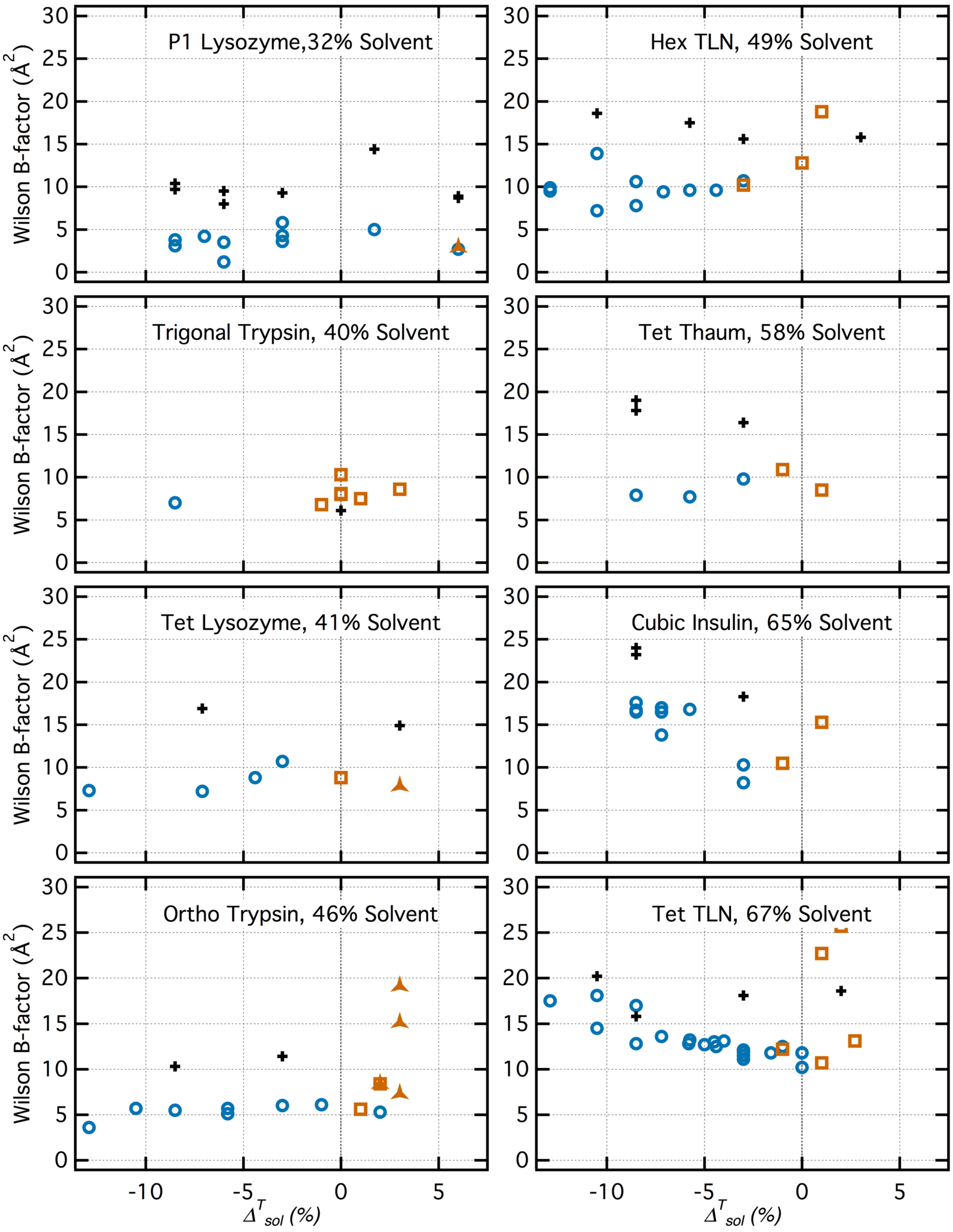

Figure S5 Wilson B-factor vs solvent contraction for all 8 crystal tested. Blue circles = LT, no ice; red squares = LT, cubic ice; red stars = LT, hexagonal ice; crosses = RT

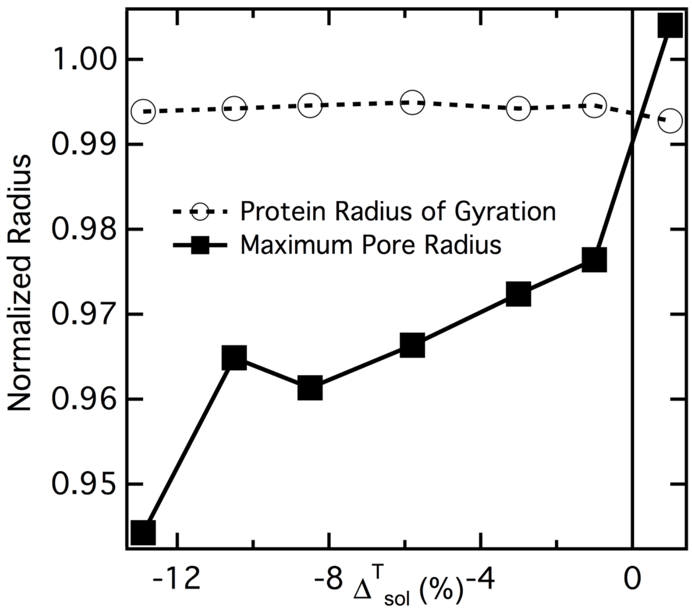

Figure S6 Maximum pore radius and thermolysin alpha carbon Rg (radius of gyration) at LT vs Δ^T_{sol}. The radii are averaged to average room temperature values. The channel contracts more than the protein for most solvent contractions.

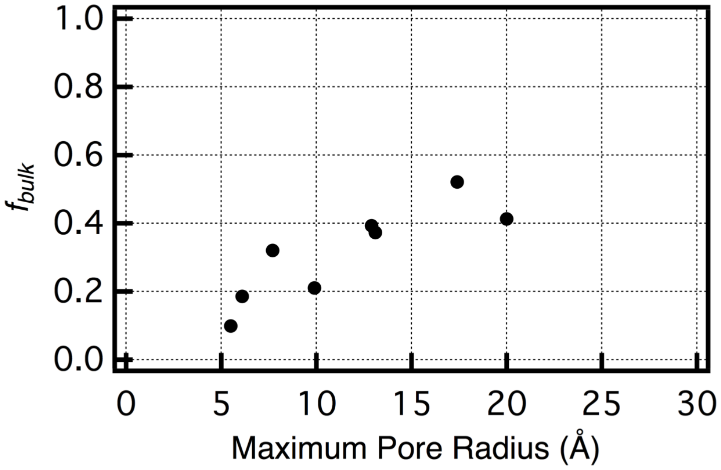

Figure S7 f_{bulk} vs pore radius. Fraction of pore solvent that behaves as bulk, according to fits of equations (1) & (2) to data in Fig 4a, assuming constant $\Delta_{prot} = -0.013$. Each plotted point represents one of the eight crystals tested. The maximum pore radius was determined with MAP_CHANNELS from refined coordinates (see Table 1) and is the radius of the largest spherical object that can fit inside the solvent channels of the crystal.

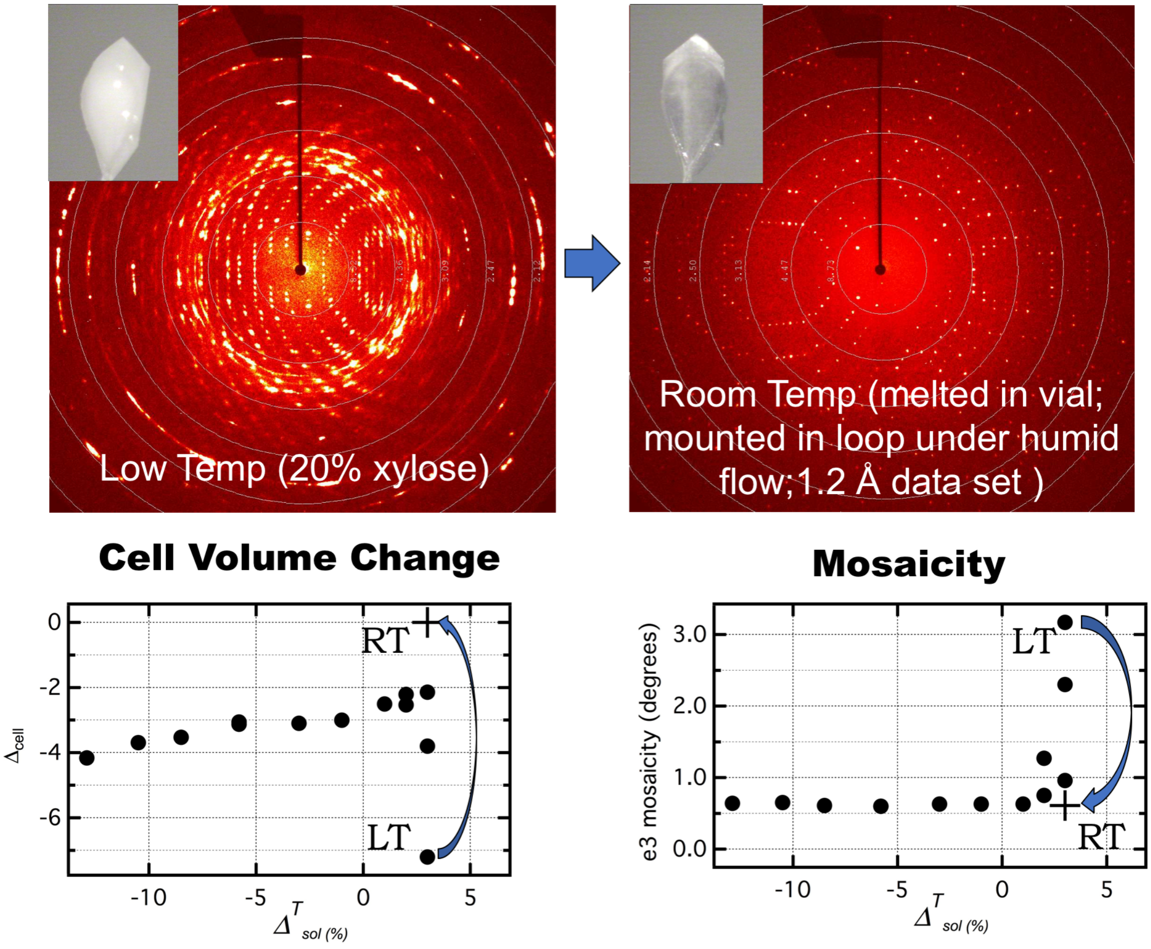

Figure S8 Reversibility of cooling induced damage with ice formation. An orthorhombic trypsin trypsin crystal (47% solvent, 10 Å radius channels) cooled with 20% xylose was visually opaque with hexagonal ice (top left), a cell volume contraction of 7% (far below the typical value of 4%) and e3 mosaicity = 3 degrees. Upon rewarming the crystal recovered (top right) to normal RT values of cell volume and mosaicity, and a data set was collected to 1.2 Å resolution. The graphs on the bottom show cell volumes and mosaicity of this annealed crystal in the context of the other trypsin crystals tested.

Table S1 Data from all crystals analysed. See spreadsheet. Temperature: data collection temperature in K; e1,e2,e3: mosaicities reported by CrysalisPro in degrees; IceType: c=cubic; h=hexagonal; m=mixed; e=powder rings not from ice;UCA,UCB,UCC: unit cell edges in Å; Vcell = unit cell volume in $Å^3$; PV = protein volume in $Å^3$;Pre/full = whether the experiment was a pre-experiment or full data set; Bfactor = Wilson B-factor ($Å^2$); Isig = <I/σ(I)> in the 2.1-2.0 Å resolution bin; Δt = exposure time (seconds); ExpType: a=aqueous, o=oil, b=both(i.e. a control for the oil experiments)

Table S2 Fractional specific volume changes for cryosolutions used. D = directly measured using a buoyancy based technique . I = interpolated. e = estimated (see Methods). Also shown are the cryosolutions used with each protein crystal.

Cryosolution	Δ_{sol}	Method	P1Lyz	TrigTrp	TetTLN	OrthoTrp	HTLN	Thm	Ins	TTLN
50% etoh	-0.132	D				x	x			x
50% meoh	-0.129	D			x	x	x			x
50% DMF	-0.105	D				x	x			x
50% MPD	-0.085	D	x	x	x	x	x	x	x	x
40% MPD	-0.072	I								x
12.5% xyl/37.5% MPD	-0.072	I					x		x	
50% PEP426	-0.072	D							x	
45% P200/5% NaCl	-0.071	e			x					
28% MPD	-0.070	I	x							
37.5% dmso	-0.060	I	x							
25/25 MPD/xylose	-0.058	I				x	x	x	x	x
30% MPD	-0.050	I								x
27.5% MPD	-0.045	I								x
50/45 % glycerol	-0.044	D;e			x					x
12.5/37.5 MPD/xylose	-0.044	I					x			
40% glycerol	-0.040	I								x
50/47/45%xylose	-0.030	D;e	x		x	x	x	x	x	x
50% glucose	-0.027	D								x
21% MPD	-0.016	I								x
40% xylose	-0.010	I				x	x	x	x	x
37.5 % glycerol	0.000	I								x
25% MPD	0.000	I								x
30% xylose/5% NaCl	0.000	e			x					
30% xylose	0.010	I		x		x	x	x	x	x
23.5% xylose	0.017	I	x							
35% glycerol	0.020	I								x
25% xylose	0.020	I				x			x	
29% glycerol	0.027	I								x
30% glycerol	0.028	I								x
20% xylose (wicked)	0.030	I				x	x	x	x	
15% xylose/5% NaCl	0.030	e			x					
15% xylose	0.040	I					x			
20% MPD	0.042	I								x
17.5% MPD	0.046	I								x
15% MPD	0.050	I								x
10% xylose	0.050	I					x			
13.5% MPD	0.054	I								x
5% xylose	0.060	I					x			
0.3 M NaNO3	0.060	e	x							
water	0.070	D					x			
Mineral Oil	-0.113	D	x	x			x			x
Infineum V8512	-0.091	D	x	x			x			x
Type A	-0.089	D		x						x
Paratac	-0.086	D								
Type B	-0.082	D		x						x
Type NVH	-0.069	D	x	x			x			x
Fomblin YR1800	-0.050	D	x	x			x			x
Santovac				x						

Table S3 Densities of external cryo-oils at 77K (from volume displacement measurements), 295 K, and calculated fractional specific volume changes.

Oil	ρ_{LT}	ρ_{RT}	Δ_{sol}
Paratone (Infineum V8512)	0.9659 (21)	0.8780[a]	-0.0910 (2)
Paratac	0.9610 (10)	0.8780[a]	-0.0863 (1)
Paraffin Oil	0.9530 (9)	0.8450[b]	-0.1133 (1)
Type A	1.0131 (17)	0.9230[a]	-0.0889 (2)
Type B	1.0053 (13)	0.9230[a]	-0.0819 (1)
Type NVH	0.9867 (19)	0.9190[a]	-0.0686 (2)
Fomblin YR1800	2.0206 (68)	1.9200[a]	-0.0498 (7)

[a]From manufacturer. [b] Measured using a 10 mL volumetric flask.

Table S4 X-ray data collection and processing for tetragonal thermolysin structures. In all cases, Space Group = $P4_12_12$, Crystal-detector distance = 65 mm, rotation range per image = 0.5°. Values for the outer shell are given in parentheses.

PDB Code	5UN3	5UU7	5UU8	5UU9	5UUA	5UUB	5UUC	5UUD	5UUE
Cryosolution	50% xylose	50% MPD	30% xylose	40% xylose	50% xylose	25%xylose/ 25%MPD	50% MPD	50% DMF	50% MeOH
Temp (K)	294	294	100	100	100	100	100	100	100
Total rotation range (°)	192	196	114	190	926	426	620	434	328
Image exp. Time (s)	60	85	90	90	60	90	90	110	60
a=*b*, *c* (Å)	97.66,108.07	98.03, 107.52	97.50, 107.92	96.82, 106.90	96.71, 106.73	96.71, 105.61	96.55, 105.28	96.44, 105.53	96.15, 104.80
Mosaicity, e3 (°)	0.61	0.61	1.41	0.60	0.60	0.62	0.62	0.70	1.00
Res. range (Å)	20.25-1.60	20.76-1.60	20.30-2.40	20.99-1.60	21.19-1.60	20.90-1.60	20.89-1.60	20.88-1.60	20.80-1.60
Total No. of reflections (from SCALA)	291919 (26734)	304481 (27968)	86781 (12244)	283032 (26069)	1377652 (126657)	627612 (57771)	904280 (83626)	637639 (58475)	471295 (43526)
No. of unique reflections (anom merged)	68814 (9901)	68909 (9817)	20765 (2966)	66699 (9594)	67217 (9666)	66508 (9551)	65939 (9478)	66120 (9523)	64281 (8796)
No. of unique reflections	121418	131139	31571	125129	127553	126191	125186	125444	121775
Completeness (%)	99.4 (99.2)	99.3 (98.4)	99.2 (99.6)	99.9 (98.9)	100.0 (100.0)	99.9 (99.9)	99.8 (99.7)	100.0 (100.0)	98.5 (93.8)
Redundancy	4.2 (2.7)	4.4 (2.8)	4.2 (4.1)	4.2 (2.7)	20.5 (13.1)	9.4 (6.0)	13.7 (8.8)	9.6 (6.1)	7.3 (4.9)
Anom Completeness	84.3 (71.1)	99.5 (98.4)	83.2 (83.7)	96.3 (88.1)	100.0 (100.0)	99.9 (99.8)	99.8 (99.8)	100.0 (100.0)	98.2 (92.4)
Anom Redundancy	2.4 (1.6)	2.3 (1.5)	2.2 (2.1)	2.2 (1.5)	10.3 (6.6)	4.7 (3.1)	7.1 (4.5)	5.0 (3.1)	3.8 (2.6)
$\langle I/\sigma(I)\rangle$ #	10.1 (0.5)	14.7 (1.1)	5.8 (1.4)	14.4 (1.4)	29.3 (2.2)	24.5 (1.9)	27.6 (2.1)	20.1 (1.4)	13.1 (0.3)
R_{meas}	0.083 (2.277)	0.056 (1.230)	0.196 (1.130)	0.058 (0.878)	0.077 (1.343)	0.059 (0.998)	0.065 (1.051)	0.064 (1.270)	0.102 (6.071)
$CC_{1/2}$	0.998 (0.258)	0.999 (0.536)	0.985 (0.761)	0.999 (0.671)	0.999 (0.734)	0.999 (0.688)	1.000 (0.752)	0.999 (0.606)	0.998 (0.178)
Overall *B* factor from Wilson plot ($Å^2$)	18.1	15.8	22.7	10.2	11.9	12.7	12.8	14.5	18.1

All crystals were collected to 1.6 Å, except 30% xylose, for which the resolution cutoff was based on $CC_{1/2}$ and the behavior of the refinement.

Table S5 Structure solution and refinement for tetragonal thermolysin structures. Values for the outer shell are given in parentheses.

Res range (Å)	20.2-1.6	20.8-1.6	20.3-2.5	21.0-1.6	21.0-1.6	20.9-1.6	19.9-1.6	19.9-1.6	20.8-1.6
Completeness (%)	98.9 (93)	99.2 (95.7)	98.4 (99.0)	97.6 (90.9)	100.0 (99.9)	100.0 (99.2)	99.8 (99.4)	100.0 (99.8)	98.3 (90.4)
No. of reflections, working set	117720 (4040)	127011 (4374)	17702 (4356)	121310 (4215)	123681 (4595)	122352 (4479)	121373 (4519)	121621 (4542)	118070 (4172)
No. of reflections, test set	3661 (136)	3983 (141)	548 (136)	3770 (126)	3770 (140)	3802 (143)	3778 (140)	3780 (142)	3653 (112)
Final R_{cryst}	0.161	0.144	0.229	0.159	0.145	0.147	0.141	0.166	0.201
Final R_{free}	0.181	0.156	0.289	0.178	0.166	0.165	0.166	0.193	0.237
No. of non-H atoms	2821	2818	2686	3075	3123	2973	2980	3017	2926
Protein	2511	2514	2474	2568	2568	2527	2523	2548	2490
Ion	15	15	6	7	14	14	18	13	13
Ligand	50	36	50	84	80	72	48	50	16
Water	245	253	156	416	461	360	391	406	407
No. of waters/no. of cryo molecules	49	84	31	52	58	45	65	41	51
R.m.s. deviations									
Bonds (Å)	0.007	0.008	0.002	0.009	0.010	0.009	0.016	0.006	0.009
Angles (°)	0.885	0.914	0.409	0.967	1.078	0.967	1.298	0.789	0.951
Avg B factors (Å^2)	24.3	24.0	27.3	20.9	20.5	21.0	20.3	23.2	25.6
Protein	21.9	21.9	26.6	18.0	17.0	18.3	17.5	20.9	23.2
Ion	34.1	27.2	26.3	13.6	26.9	24.1	22.7	26.1	26.9
Ligand	54.2	53.8	47.5	38.1	40.0	39.8	39.4	36.5	33.4
Water	41.6	41.2	30.2	35.5	37.0	36.8	36.1	36.0	40.0
Ramachandran plot									
Most favoured (%)	96.0	95.6	94.7	95.8	95.5	95.8	95.2	94.5	95.0
Allowed (%)	2.7	2.7	3.6	2.8	2.8	2.8	3.0	3.8	3.3

Table S6 X-ray data collection and processing for orthorhombic trypsin structures. In all cases, Space Group = $P2_12_12_1$, Crystal-detector distance = 61 mm, rotation range per image = 0.5° Values for the outer shell are given in parentheses.

PDB Code	6AVL	6B6N	6DZF	6B6O	6B6P	6B6Q	6B6R	6B6S	6B6T
Cryosolution	50% xylose	50% MPD	20% xylose	20% xylose	30% xylose	50% xylose	50% MPD	50% EtOH	50% MeOH
Temp (K)	294	294	100	100	100	100	100	100	100
Total rotation range (°)	98	86	103	87	86	86	105	75	102
Exposure time per image (s)	5	5	10	10	10	10	10	5	10
a (Å)	54.87	54.69	54.12	54.44	54.65	54.43	54.12	54.31	54.38
b (Å)	58.76	58.54	56.84	58.17	58.31	58.27	58.19	58.05	58.10
c (Å)	67.48	,67.58	65.48	65.94	66.41	66.32	66.49	66.25	65.85
Mosaicity (°)	0.62	0.60	3.17	2.30	0.63	0.63	0.61	0.75	0.64
Res range (Å)	13.8 – 2.0	13.2-2.0	13.5 – 2.2	13.2 – 2.4	13.8 – 2.0	13.8-2.0	13.9-2.0	13.7 – 2.0	13.7 – 2.0
Total No. of reflections	55495 (7602)	49686 (6856)	36955 (5042)	27012 (3970)	48536 (6651)	48258 (6591)	58789 (7954)	41402 (5633)	54248 (7733)
No. of unique reflections	15148 (2193)	15002 (2175)	10574 (1493)	8437 (1244)	14610 (2092)	14616 (2102)	14445 (2046)	14509 (2035)	14481 (2031)
Completeness (%)	99.2 (99.2)	99.0 (99.1)	98.8 (98.2)	98.4 (100.0)	98.4 (98.2)	98.9 (99.9)	98.5 (97.4)	99.0 (97.9)	98.9 (97.4)
Redundancy	3.7 (3.5)	3.3 (3.2)	3.5 (3.4)	3.2 (3.2)	3.3 (3.2)	3.3 (3.1)	4.1 (3.9)	2.9 (2.8)	3.9 (3.8)
$\langle I/\sigma(I) \rangle$	5.4 (2.8)	17.1 (7.4)	3.3 (1.1)	3.9 (2.6)	28.4 (16.1)	26.7 (14.5)	29.0 (15.2)	18.3 (9.8)	28.8 (17.0)
R_{meas}	0.155 (0.364)	0.059 (0.164)	0.398 (0.995)	0.28 (0.35)	0.033 (0.066)	.035 (.073)	0.039 (0.083)	0.048 (0.109)	0.036 (0.067)
$CC_{1/2}$	0.98 (0.87)	1.00 (0.97)	0.61 (0.34)	0.79 (0.89)	1.00 (1.00)	1.00 (0.99)	1.00 (1.00)	1.00 (0.99)	1.00 (1.00)
Overall *B* factor from Wilson plot ($Å^2$)	11.4	10.3	19.1	15.1	5.6	6.0	5.5	5.1	3.9

Table S7 Structure solution and refinement for orthorhombic trypsin structures.

Resolution range (Å)	13.5 – 2.0	13.2 – 2.0	13.5 – 2.2	13.0 – 2.4	13.4 – 2.0	13.3 – 2.0	12.9 – 2.0	12.9 – 2.0	13.2 – 2.0
Completeness (%)	99.2 (99.2)	98.9 (98.8)	97.3 (93.0)	98.3 (99.6)	98.4 (97.9)	99.0 (99.8)	98.4 (97.9)	99.0 (97.6)	99.1 (97.6)
No. of reflections, working set	14319 (2358)	14189 (2327)	9847 (2316)	7955 (2633)	13796 (2238)	13806 (2740)	13660 (2689)	13710 (2663)	13676 (2660)
No. of reflections, test set	796 (124)	788 (120)	544 (122)	451 (157)	772 (120)	765 (146)	753 (146)	758(144)	760 (142)
Final R_{cryst}	0.150	0.121	0.303	0.240	0.122	0.119	0.120	0.121	0.122
Final R_{free}	0.205	0.157	0.384	0.345	0.171	0.170	0.172	0.176	0.173
No. of non-H atoms	1874	1890	1754	1894	1970	1967	1947	1997	1980
Protein	1672	1679	1647	1659	1681	1688	1661	1689	1665
Ion	6	6	6	11	11	11	16	16	11
Ligand	29	25	19	29	29	29	25	45	49
Water	167	180	82	195	249	239	245	247	255
No. of waters/no. of cryo molecules	84	88	100	100	125	120	122	22	13
R.m.s. deviations									
Bonds (Å)	0.007	0.002	0.002	0.007	0.008	0.007	0.008	0.008	0.007
Angles (°)	0.871	0.520	0.517	0.928	0.950	0.848	0.859	0.902	0.861
Avg *B* factors (Å^2)	17.3	15.7	25.4	17.5	11.0	11.0	10.4	12.1	10.0
Protein	15.9	14.1	25.7	17.1	9.2	9.3	8.8	10.4	8.3
Ion	54.0	59.0	68.7	42.6	50.2	48.0	42.2	34.8	49.0
Ligand	23.5	21.6	33.9	30.8	28.8	24.2	11.6	19.5	17.8
Water	28.5	29.0	15.2	17.1	19.0	20.4	18.8	21.3	17.8
Ramachandran plot									
Most favoured (%)	97.0	97.0	94.4	93.7	97.5	97.7	97.1	96.9	98.1
Allowed (%)	3.0	3.0	5.2	6.3	2.5	2.1	2.9	2.6	1.9

Table S8 X-ray data collection and processing for triclinic lysozyme structures. In all cases, Space Group = P1, Crystal-detector distance = 61 mm, rotation range per image = 1.0° Values for the outer shell are given in parentheses.

PDB Code	6D6E	6D6G	6D6F	6D6H
Cryosolution	47% xylose	47% MPD	47% xylose	47% MPD
Temp (K)	294	294	100	100
Total rotation range (°)	290	221	284	248
Exposure time per image (s)	20	10	15	10
a,*b*, *c* (Å)	27.27,32.24,34.32	27.33, 32.15, 34.32	26.96, 31.86, 33.99	26.99, 31.69,33.99
α,β,γ (o)	88.22,71.18,68.69	88.24, 70.78,68,69	88.23, 71.54, 68.41	88.27,71.25,68.50
Mosaicity,e3 (°)	1.05	1.02	1.08	1.07
Resolution range (Å)	13.67-2.00	13.61-2.00	13.56-2.00	13.50-2.00
Total No. of reflections (from SCALA)	20266 (2747)	15372 (2090)	19221 (2627)	16753 (2300)
No. of unique reflections	6847 (997)	6714 (994)	6647 (958)	6475 (960)
Completeness (%)	98.9 (97.9)	97.3 (98.2)	99.2 (98.5)	97.1 (98.4)
Redundancy	3.0 (2.8)	2.3 (2.1)	2.9 (2.7)	2.6 (2.4)
$\langle I/\sigma(I) \rangle$	14.5 (5.2)	13.1 (4.4)	16.9 (7.8)	18.7 (9.0)
R_{meas}	0.071 (0.241)	0.072 (0.278)	0.058 (0.162)	0.058 (0.878)
$CC_{1/2}$	0.997 (0.942)	0.997 (0.909)	0.998 (0.974)	0.999 (0.671)
Overall *B* factor from Wilson plot (Å^2)	9.9	9.7	5.8	3.8

Table S9 Structure determination and refinement statistics for triclinic lysozyme structures.

Resolution range (Å)	13.4 – 2.0	13.6-2.0	13.6 – 2.0	13.2 – 2.0
Completeness (%)	99.1 (99.0)	97.5 (98.6)	99.5 (99.0)	97.3 (98.6)
σ cutoff				
No. of reflections, working set	6519 (3232)	6398 (3204)	6331(3115)	6166 (3092)
No. of reflections, test set	320 (181)	310 (175)	312 (175)	304 (172)
Final R_{cryst}	0.134	0.132	0.137	0.125
Final R_{free}	0.188	0.182	0.200	0.192
Cruickshank DPI				
No. of non-H atoms	1129	1117	1149	1200
Protein	1005	993	998	1015
Ion	24	24	28	28
Ligand	20	20	30	36
Water	80	80	93	121
No. of waters/no. of cryo molecules	-	-	93	60
R.m.s. deviations				
Bonds (Å)	0.002	0.002	0.002	0.002
Angles (°)	0.548	0.501	0.549	0.521
Average *B* factors (Å^2)	15.9	18.3	13.5	10.0
Protein	15.9	16.7	12.0	8.6
Ion	31.2	29.1	21.6	14.8
Ligand	48.5	50.4	36.4	32.0
Water	26.9	27.1	19.7	15.1
Ramachandran plot				
Most favoured (%)	98.4	98.4	96.8	97.5
Allowed (%)	1.6	1.6	3.2	2.5

Table S10 X-ray data collection and processing for hexagonal thermolysin structures. Space Group = $P6_122$, crystal to detector distance = 65 mm (except for 20% xylose – 107.24 mm), rotation range per image = 0.5° Values for the outer shell are given in parentheses.

PDB Code	6D5N	6D5O	6D5P	6D5Q	6D5R	6D5S	6D5T	6D5U
Cryosolution	50% xylose	50% DMF	20% xylose	30% xylose	50% xylose	50% MPD	50% MPD	50% MeOH
Temp (K)	294	294	100	100	100	100	100	100
Total rotation range (°)	54	57	69	69	72	66	60	53
Exposure time per image (s)	40	20	40	30	40	30	30	40
Detector distance (mm)	65.0	65.0	107.2	65.0	65.0	65.0	65.0	65.0
a=b, c (Å)	93.80,132.29	93.93,131.66	93.31,129.13	93.39,129.88	92.92,130.12	92.96,128.83	92.79,128.87	92.81,127.7
Mosaicity (°)	0.57	0.57	1.27	0.99	0.66	0.65	0.61	0.80
Resolution range (Å)	13.85-2.00	13.97-2.00	13.75-3.00	13.83-2.00	13.80-2.0	13.81-2.00	13.74-2.00	13.92-2.00
Total No. of reflections (from SCALA)	136554 (14757)	144893 (15510)	52731 (7444)	201052 (22106)	188157 (20415)	161611 (17453)	131995 (12311)	127362 (13804)
No. of unique reflections	23812 (3384)	23548 (3301)	6950 (994)	23112 (3325)	23018 (3300)	22529 (3228)	22665 (3220)	22314 (3233)
Completeness (%)	99.6 (99.6)	99.0 (97.8)	98.5 (99.9)	99.4 (99.9)	99.7 (100.0)	98.9 (98.8)	99.5 (98.9)	99.2 (99.9)
Redundancy	5.7 (4.4)	6.2 (4.7)	7.6 (7.5)	8.7 (6.6)	8.2 (6.2)	7.2 (5.4)	5.8 (3.8)	5.7 (4.3)
$\langle I/\sigma(I)\rangle$	11.7 (2.3)	8.5 (2.3)	5.1 (3.0)	6.8 (0.7)	17.9 (4.7)	15.2 (4.4)	13.7 (3.2)	5.2 (1.7)
R_{meas}	0.142 (0.745)	0.154(0.725)	0.324 (0.469)	0.305 (2.372)	0.104 (0.417)	0.120 (0.425)	0.117 (0.490)	0.260 (0.877)
$CC_{1/2}$	0.995 (0.736)	0.993 (0.760)	0.892 (0.922)	0.981 (0.336)	0.998 (0.922)	0.996 (0.903)	0.996 (0.833)	0.976 (0.601)
Overall B factor from Wilson plot (Å^2)	19.4	18.6	27.1	18.8	10.7	7.8	10.6	9.5

Table S11 Structure determination and refinement statistics for hexagonal thermolysin structures.

Resolution range (Å)	13.4 – 2.0	13.6 - 2.0	13.8 – 3.0	13.8 – 2.0	13.8-2.0	12.9 - 2.0	13.7 - 2.0	13.9 - 2.0
Completeness (%)	99.8 (99.4)	98.9 (96.9)	97.9 (98.6)	94.8 (97.9)	99.9 (100.0)	98.8 (98.5)	99.7 (98.9)	99.0 (99.9)
No. of reflections, working set	22620 (2753)	22360 (2683)	6535(3209)	20938(3031)	21882 (2690)	21451 (2627)	21558 (2615)	21235 (2616)
No. of reflections, test set	1125 (118)	1126 (130)	324 (153)	1030 (130)	1086 (117)	1063 (118)	1074 (116)	1057 (118)
Final R_{cryst}	0.150	0.154	0.232	0.217	0.154	0.144	0.153	0.167
Final R_{free}	0.188	0.193	0.307	0.299	0.204	0.190	0.196	0.223
No. of non-H atoms								
Protein	2463	2492	2444	2455	2509	2534	2479	2499
Ion	5	5	5	5	5	5	5	5
Ligand	37	37	47	37	57	33	25	37
Water	140	135	146	239	293	305	239	323
Total	2645	2669	2642	2736	2864	2877	2748	2864
No. of waters/no. of cryo molecules	70	34	49	120	73	153	239	32
R.m.s. deviations								
Bonds (Å)	0.002	0.002	0.002	0.002	0.002	0.003	0.004	0.004
Angles (°)	0.55	0.49	0.47	0.57	0.55	0.61	0.76	0.63
Average B factors (Å^2)	20.7	22.2	30.2	31.1	18.0	14.1	15.7	19.3
Protein	19.8	21.4	31.0	30.5	16.4	12.8	14.9	18.2
Ion	16.6	17.2	26.7	29.0	13.7	11.2	15.2	16.2
Ligand	46.3	42.0	49.5	45.9	42.5	32.3	32.4	27.6
Water	29.9	30.8	11.2	36.3	27.6	23.4	23.0	27.2
Ramachandran plot								
Most favoured (%)	95.1	95.3	94.5	95.1	94.4	94.3	93.9	94.2
Allowed (%)	3.3	3.0	3.5	3.0	3.9	3.9	4.4	3.8

Table S12 Data Collection statistics for alpha-lactalbumin crystals "A" and "E" from the text. Crystal A was one of the first data sets collected, and data set E was the final data set collected after cryo-optimization using cryosolvent thermal contraction as a guide.

Cryocondition from text	A	E
Cryosolution	25 % glycerol	10% MPD/25% MeOH
Temp (K)	100	100
Total rotation range (°)	124	406
Exposure time per image (s)	60	90
a,*b*, *c* (Å)	70.3,102.1,117.0	71.9,104.1,115.7
Mosaicity (°)	1.74	0.62
Resolution range (Å)	30.7-2.8	29.55-1.65
Total No. of reflections	100240 (14446)	814645 (87658)
No. of unique reflections	21266 (3072)	104673 (15009)
Completeness (%)	99.3 (99.5)	99.9 (99.2)
Redundancy	4.7 (4.7)	7.8 (5.9)
$\langle I/\sigma(I)\rangle$	9.8 (1.2)	18.9 (1.4)
R_{meas}	0.158 (1.509)	0.061 (1.280)
$CC_{1/2}$	0.995 (0.536)	1.00 (0.55)
Overall *B* factor from Wilson plot (Å^2)	45.8	17.7

References

Frauenfelder, H., Hartmann, H., Karplus, M., Kuntz, I. D., Jr., Kuriyan, J., Parak, F., Petsko, G. A., Ringe, D., Tilton, R. F., Jr., Connolly, M. L. & et al. (1987). *Biochemistry* **26**, 254-261.

Juers, D. H. & Matthews, B. W. (2001). *J. Mol. Biol.* **311(4)**, 851-862.

Li, A. J. & Nussinov, R. (1998). *Proteins: Struct., Funct., Genet.* **32**, 111-127.
